# cfDNA UniFlow: a unified preprocessing pipeline for cell-free DNA data from liquid biopsies

**DOI:** 10.1093/gigascience/giae102

**Published:** 2024-12-20

**Authors:** Sebastian Röner, Lea Burkard, Michael R Speicher, Martin Kircher

**Affiliations:** Berlin Institute of Health (BIH) at Charité–Universitätsmedizin Berlin, 10178 Berlin, Germany; Berlin Institute of Health (BIH) at Charité–Universitätsmedizin Berlin, 10178 Berlin, Germany; University of Potsdam, Institute for Biochemistry and Biology, 14469 Potsdam, Germany; Institute of Human Genetics, Diagnostic and Research Center for Molecular BioMedicine, Medical University of Graz, 8010 Graz, Austria; Berlin Institute of Health (BIH) at Charité–Universitätsmedizin Berlin, 10178 Berlin, Germany; Institute of Human Genetics, University Medical Center Schleswig-Holstein, University of Lübeck, 23562 Lübeck, Germany

**Keywords:** cell-free DNA, liquid biopsies, sequence analysis, cancer detection, workflow

## Abstract

**Background:**

Cell-free DNA (cfDNA), a broadly applicable biomarker commonly sourced from urine or blood, is extensively used for research and diagnostic applications. In various settings, genetic and epigenetic information is derived from cfDNA. However, a unified framework for its processing is lacking, limiting the universal application of innovative analysis strategies and the joining of data sets.

**Findings:**

Here, we describe cfDNA UniFlow, a unified, standardized, and ready-to-use workflow for processing cfDNA samples. The workflow is written in Snakemake and can be scaled from stand-alone computers to cluster environments. It includes methods for processing raw genome sequencing data as well as specialized approaches for correcting sequencing errors, filtering, and quality control. Sophisticated methods for detecting copy number alterations and estimating and correcting GC-related biases are readily incorporated. Furthermore, it includes methods for extracting, normalizing, and visualizing coverage signals around user-defined regions in case-control settings. Ultimately, all results and metrics are aggregated in a unified report, enabling easy access to a wide variety of information for further research and downstream analysis.

**Conclusions:**

We provide an automated pipeline for processing cell-free DNA sampled from liquid biopsies, including a wide variety of additional functionalities like bias correction and signal extraction. With our focus on scalability and extensibility, we provide a foundation for future cfDNA research and faster clinical applications. The source code and extensive documentation are available on our GitHub repository (https://github.com/kircherlab/cfDNA-UniFlow).

## Introduction

**Figure 1:**
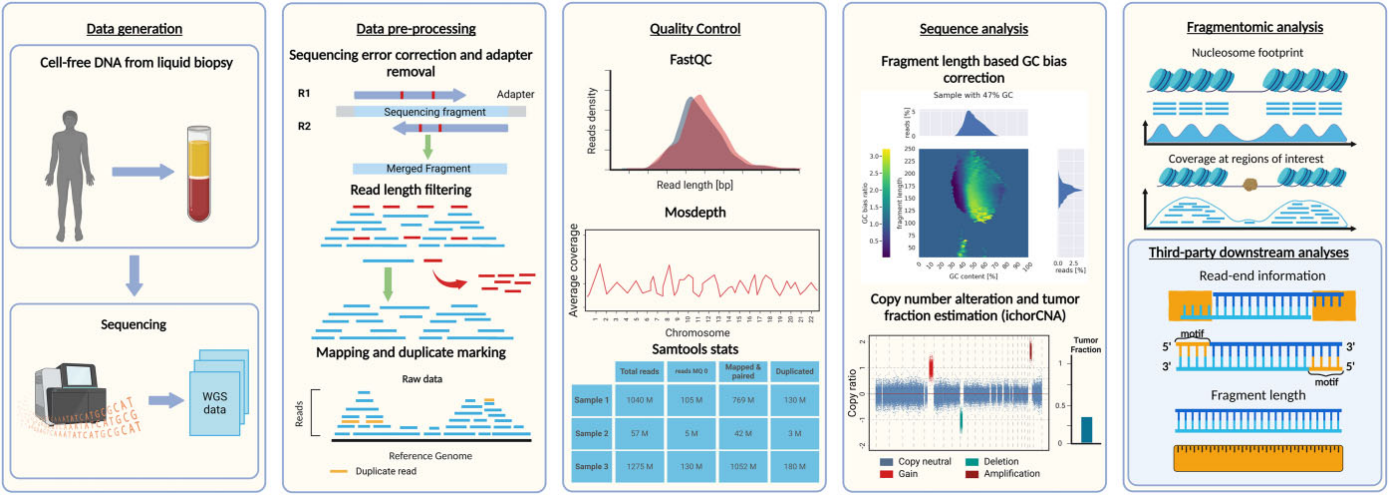
Overview of cfDNA analysis. The leftmost panel depicts data generation by liquid biopsy sampling followed by library preparation and sequencing. The second panel shows the entry point of cfDNA Uniflow. It displays the core functionality of merging reads/removing adapters, length filtering, mapping to a reference genome, and duplicate marking. Sample quality control is shown in the third panel and, for example, performed using FastQC, Mosdepth, and SAMtools stats. The fourth panel shows optional steps of GC bias correction and estimation of copy number alterations and tumor proportion. Finally, results are aggregated, for example, in a report and used for downstream analyses (fifth panel). Figure created with BioRender.

Cell-free DNA (cfDNA) is found in many bodily fluids like blood plasma and urine [1]. It is believed to be primarily derived from natural degradation processes during cell turnover [2]. However, the proportion of cell types and tissues contributing to cfDNA changes in the context of certain physiological conditions or disease processes [3, 4]. Thus, signals in cfDNA might serve as relevant biomarkers in health and disease. Collecting cfDNA in so-called liquid biopsies (Fig. 1) is considered noninvasive and led to an increased research interest in the biomedical field for using cfDNA in allograft (i.e., donor organ) rejection, prenatal testing and diagnostics, and disease detection and health monitoring [5] (especially for cancer).

Over the past years, many approaches have been developed to extract information from cfDNA samples for various applications. Methods include identifying allelic differences at known disease markers, detecting and tracking mutations [6] and copy number alterations (CNAs) in tumor cells [7], analyzing DNA fragmentation differences [3, 8, 9], and measuring methylation state [10–12]. While these methods exploit different signals, all rely on the precise quantification of read distributions, and slight changes in read recovery affect their results (Fig. 1).

Therefore, consistent data quality is the primary requirement for developing these new diagnostic methods (Fig. 1). Even though sample handling is constantly streamlined, individual differences of sample donors and logistic factors like time of sample collection, duration, conditions of storage, and further preanalytical handling are challenging to fully control in a clinical context but have been shown to affect the quality of cfDNA samples [13–16]. Additionally, detecting signals of interest (e.g., from circulating tumor DNA, ctDNA) in a background mainly derived from hematopoietic cells [17] is not trivial, emphasizing the need for optimal data quality.

One way to mitigate some preanalytical effects and technical biases introduced during sequencing of cfDNA samples is to include specialized correction and sampling steps during computational processing of the data (Fig. 1). Even though the need has been identified previously in the field of cfDNA, community standards are still lacking for preprocessing genome sequencing data from cfDNA [7, 8, 18–23].

One reason for the lack of dedicated cfDNA pipelines might be that many publications in the field are focused on the downstream analysis, like the classification of disease samples, relying on unpublished in-house pipelines for data processing. Further, important correction steps are often tailored toward specific features and tightly integrated in downstream analysis pipelines, making it difficult to generalize and transfer them to new projects [7, 8]. Nevertheless, there have been some approaches trying to address the need for community standards. A notable one is the FinaleDB project, which aggregates cfDNA samples from multiple sources, processes them in a uniform manner, and provides fragment coordinates via a web portal [24]. To protect the privacy of patients, the data are anonymized during processing, removing all sequence information and making it unsuitable for analyses not focused on fragmentation patterns. Currently, this pipeline does not address issues of bias correction, which might be one of the most relevant tasks in such data aggregation efforts between different studies. The project getting closest to setting a community standard for processing samples not just for fragmentomics applications is called cfDNApipe. It combines many useful tools for basic processing of normal and bisulfite converted DNA sequences. The utility functions include generation of summary statistics, GC-bias correction tailored toward CNV detection, and extraction of a limited number of features [25]. However, the software seems to be designed for single computer use, lacking many of the features provided by a full-fledged workflow management system, making it hard to scale analysis in different environments, like compute clusters. Moreover, the design does not allow for easy integration of new functionalities, creating the need for either an additional workflow management system or extensive modification of the original code (detailed comparison available in Supplementary Table S1).

Technical biases and missing community standards cause several drawbacks for the field. First, users rely on standard processing pipelines from other fields, which might not be suitable for specific analyses. They might also feel the need to develop their own pipelines by selecting appropriate tools and tuning parameters optimized on the available set of samples. Second, it adds additional overhead when comparing across multiple studies. Here, researchers are frequently required to work with the original processing of each site, potentially introducing technical biases in the analysis. Alternatively, reprocessing data from multiple sites can reduce technical biases between studies but creates an additional computational and organizational burden (including access to raw and protected genetic data). Third, it can be hard to keep track of all sample-level information when building analysis pipelines using many samples, especially when information gets scattered across mutiple files.

To jointly address several of these problems, we developed an easy-to-use unified preprocessing workflow for cell-free DNA written in Snakemake. It combines a curated list of tools for processing genomic cfDNA samples, custom tools for reducing technical biases, and tools for estimating additional characteristics like copy number states. Our pipeline is implemented with high configurability, scalability from single computers to high-performance compute clusters, and a sophisticated reporting system.

## Overview and Implementation

### Implementation

We implemented the cfDNA UniFlow workflow in the popular workflow management system Snakemake [26]. This makes it easy to scale the workflow in different computing environments and allows for parallel processing of multiple samples. Further, most of the rules are implemented to enable multiprocessing and efficiently utilize multiple cores for each task. Conveniently, default resources like genome references or standard adapter files can be downloaded, if the workflow is not configured to point to already available resources. A detailed overview of the workflow is available in Supplementary Fig. S1. Briefly, cfDNA UniFlow covers 3 parts between data generation and downstream analysis: data preprocessing, quality control, and utility functions (Fig. 1).

### Preprocessing

**Figure 2:**
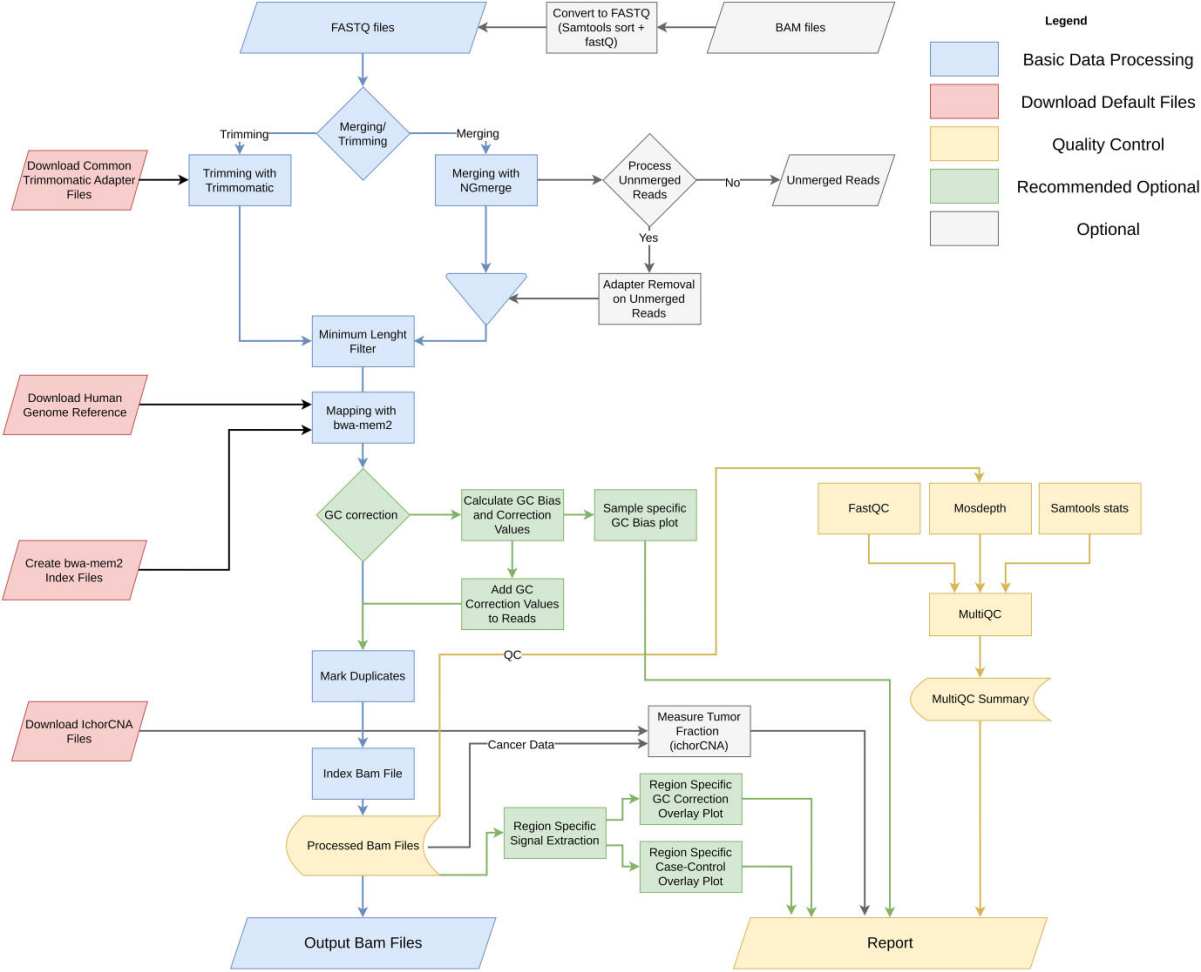
Overview of unified cfDNA preprocessing workflow. Functionalities are color-coded by task. Blue boxes contain the core functionality of cfDNA Uniflow. Red boxes represent rules for the automatic download of public resources. Yellow boxes summarize the quality control and reporting steps. Finally, gray and green boxes are optional steps, with green boxes being highly recommended.

The core preprocessing steps (Fig 2, components depicted in blue) expect FASTQ files as input. Alternatively, existing alignments (BAM files) can be provided for reprocessing. In the latter case, the workflow automatically converts these to FASTQ files using SAMtools [27]. Afterward, reads can be merged with NGmerge [28], which also removes sequencing library adapters and corrects sequencing errors and ambiguous bases based on the read-overlap consensus. Reads that were not merged can be postprocessed using NGmerge adapter removal mode and can be included in the mapping process. Alternatively, the merging step can be skipped, and reads will only be trimmed using Trimmomatic. Prior to mapping with bwa-mem2 [29], reads are further filtered based on their length, excluding reads that are shorter than a configurable threshold. Finally, duplicate reads are marked (SAMtools markdup) before the BAM files of the samples are passed to the next step.

### Quality control

In the quality control (QC) step (Fig. 2, components depicted in yellow), general postalignment statistics and graphs are calculated for each sample with SAMtools stats [27] and FastQC [30]. Additional information on sample-wide median coverage and coverage at different genomic regions is calculated via Mosdepth [31]. The QC results are aggregated in a HTML report via MultiQC [32], and an example is shown Supplementary Fig. S2.

### Signal extraction

In the last step, additional utility modules (Fig. 2, components depicted in green) can be configured and executed. This includes our in-house GC bias estimation and correction method cfDNA_GCcorrection [33], an extension of the method described by Benjamini and Speed [18]. As fragmentation in cfDNA is driven by natural degradation processes, libraries constructed from liquid biopsies tend to have fragments of a wide range of lengths and do not follow the original assumption that length is well approximated by the mean fragment length. Therefore, we estimate the expected fragment distribution by sampling regions along the reference genome, counting all possible fragments for a specified range of fragment lengths and sorting them in bins of their GC content. Afterward, we measure the sample specific fragment distribution in the same regions, scale them, and compare them to the theoretical distribution. Based on the ratio of observed and expected, we calculated correction values for each fragment length and GC content. The resulting weights are attached to the reads as tags, which can be used for a wide variety of downstream signal extraction methods, while preserving the original read coverage and fragmentation patterns. We provide specialized signal extraction routines to extract coverage derived signals using read weights. Further, we included the widely used tool ichorCNA [7] to identify copy number alterations and estimate tumor fraction. An example of the output is available in Supplementary Fig. S3.

### Reporting

Finally, all information provided by the previous steps is aggregated in a comprehensive HTML report. This includes summary statistics on workflow execution provided by Snakemake and plots and summary statistics produced in the quality control steps. Additional information from optional steps includes a general estimation of sample-specific GC bias parameters (Supplementary Fig. S4), the effects of GC bias correction in user-defined regions (Supplementary Fig. S5), and plots of copy number alterations created by ichorCNA. Finally, case-control plots are generated and included if more than 1 class of samples is provided (Supplementary Fig. S6).

## Results

To test and showcase cfDNA Uniflow, we use 3 exemplary cfDNA samples (healthy H01, breast cancer B01, prostate cancer P01) with different conditions and average GC contents from the European Genome-Phenome Archive Study EGAS00001006963. Each sample was converted to FASTQ files and processed in our pipeline with standard parameters for human reference build GRCh38/hg38. As user-defined regions of interest, we selected 10,000 binding sites of LYL1, a transcription factor (TF) associated with hematopoietic cells [34], and GRHL2, an important pioneer TF for epithelial cells [35–37] playing a role in a wide variety of cancer types [38–42]. Both TFs are especially suited due to their association with expected tissue contributions in our samples and because they have high GC content binding sites.

**Figure 3:**
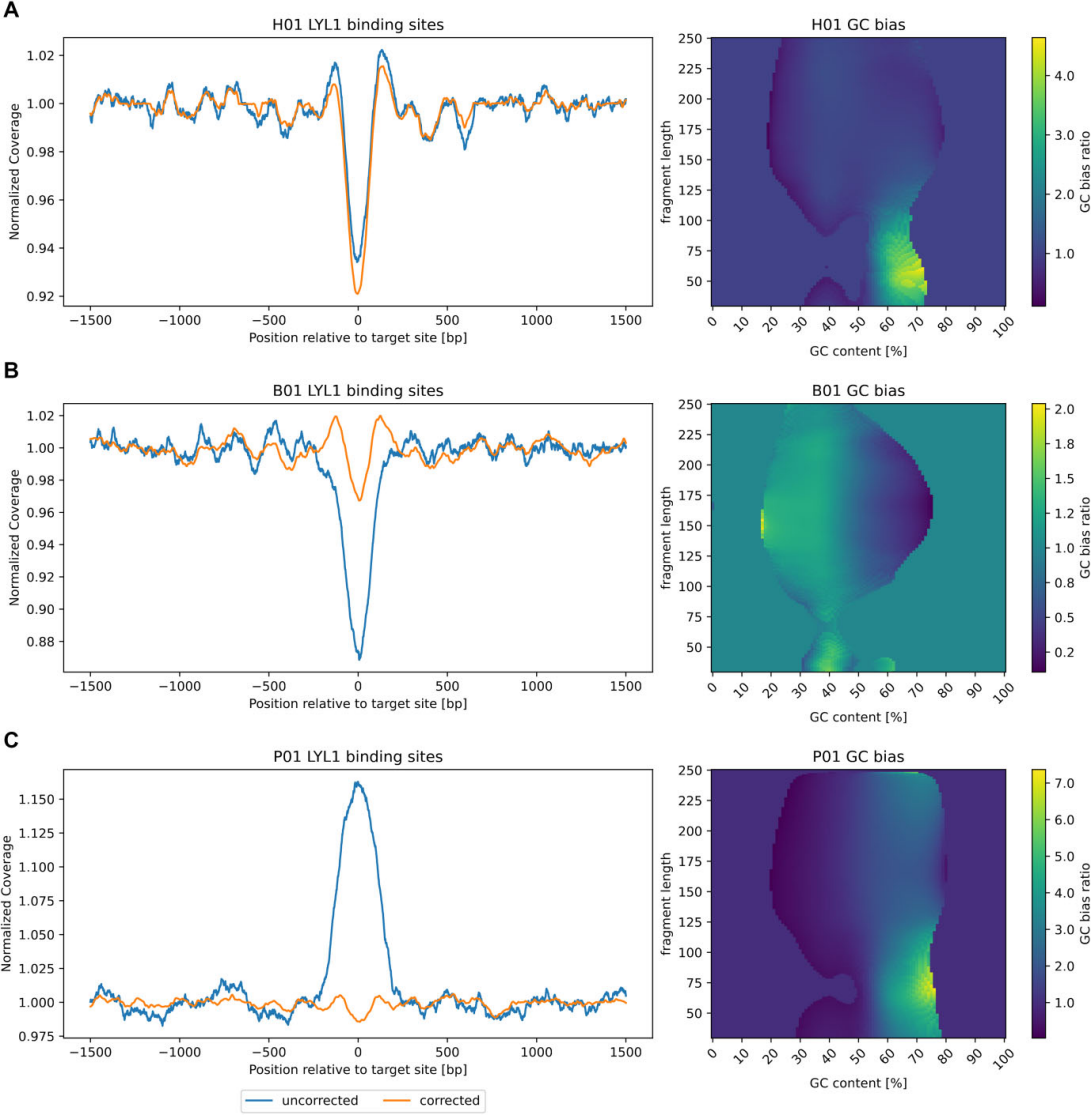
Effects of GC bias on regional and global scale. Composite coverage signals of 10,000 LYL1 transcription factor binding sites (left) and global bias profiles (right) for 3 cfDNA samples are shown. (A) Signals and profile for a healthy sample (H01) with an average GC content of 41%. The GC profile (right) is relatively balanced between observed and expected fragments. Respectively, the GC bias corrections have only minor effects on the composite coverage signal (left). (B) GC bias effects for a breast cancer sample with an average GC content of 38%. The global GC profile shows an overrepresentation of lower GC content fragments (brighter color) and an underrepresentation of higher GC content fragments (darker color). This results in an underestimation of coverage (overestimation of accessibility) at the LYL1 binding sites. After GC correction, the signal is closer to the surrounding coverage, consistent with lower relative contributions of hematopoietic cells and fewer open sites. In contrast, (C) shows the GC bias effects of a prostate cancer sample with an average GC content of 45%. The global GC profile is skewed toward a higher GC content, leading to an overestimation of coverage around the LYL1 binding sites. After GC correction, the signal is closer to the surrounding coverage, indicating lower contributions of hematopoietic cells with accessible LYL1 sites.

This can be seen in Fig. 3, which shows coverage overlays centered on LYL1 binding sites and illustrates the global and regional effects of GC biases in the respective samples. The healthy sample H01, with an average GC content of 41%, shows a balanced global GC profile (Fig.   3A), and accordingly, the GC bias correction shows almost no effects on the composite signal. We see the strongest drop of coverage at the TF binding site, expected for a sample of mainly hematopoietic origin where many LYL1 binding sites are expected to be accessible to the TF. B01, a breast cancer sample with an average GC content of 38%, shows an overrepresentation of fragments with GC content lower than the genome average and an underrepresentation of fragments with higher GC content (Fig. 3B). This leads to a distortion of the composite coverage signal around the LYL1 binding sites. Without GC correction, the drop in coverage would be overestimated. After correction, coverage at the site is closer to the coverage of the surrounding regions, consistent with an expected signal dilution compared to the healthy sample (Fig. 3A) due to a higher contribution of nonhematopoietic cell types in this cancer sample (Fig. 3B). The same should be true for sample P01 (Fig. 3C), a prostate cancer sample with an average GC content of 45%. However, the global GC bias profile (right panels) shows the inverse trend to sample B01, with a shift of fragment distribution toward a higher GC content. Unsurprisingly, the signal around the binding sites is distorted toward higher coverage prior to the GC correction (i.e., suggesting that the TF binding sites are not accessible). After GC correction, the signal looks similar to the one shown for B01, less open than the healthy sample and consistent with an increased contribution of nonhematopoietic cell types. Global effects of GC bias correction on fragment distribution and a comparison to 2 other fragment-based methods are provided in Supplementary Fig. S7.

**Figure 4:**
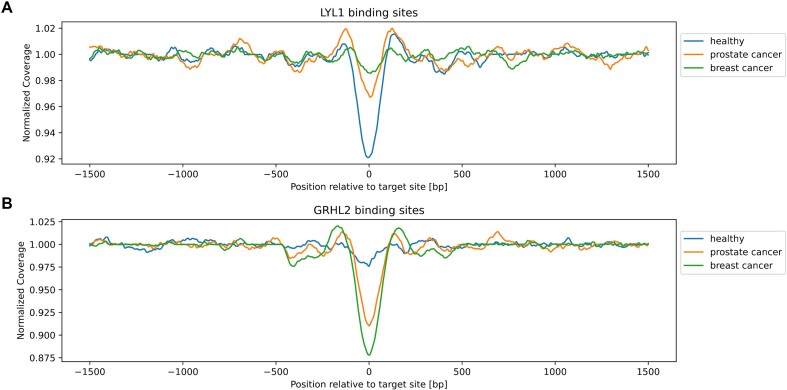
Case-control plots around GRHL2 and LYL1 binding sites. (A) GC-corrected composite coverage signals around 10,000 centered LYL1 transcription factor binding sites. The healthy sample (H01) shows lower relative coverage (i.e., higher accessibility) at the center of the binding site overlay. This is consistent with higher LYL1 activity in hematopoietic cells. In contrast, both cancer samples show higher relative coverage in the central region, in line with a higher proportion of nonhematopoietic cells contributing to the signal. (B) Composite coverage signals around 10,000 GRHL2 binding sites after GC correction. Both cancer samples show a lower central coverage compared to surrounding regions (i.e., higher accessibility), indicating higher activity than in the healthy sample. This is consistent with GRHL2 expression being associated with different cancers.

In addition to the GC bias plots for individual samples, we provide case-control plots for comparing sample classes with a control in the same plot. In our example, the healthy sample H01 would be the control, and we are comparing samples for LYL1 and GRHL2 sites. As noted, the expected signal around LYL1 binding sites is a drop in coverage for samples mainly derived from hematopoietic cells. When the contribution of nonhematopoietic cell types, in which LYL1 is not expressed, increases, we expect to see a relative increase in coverage around the binding sites. Accordingly, the signals shown for our 3 test samples (Fig. 4A) are in line with that expectation. For GRHL2, we expect the opposite signal. As healthy samples should not include many contributions from tissues with high GRHL2 activity, the expected coverage signal should be similar to the surrounding regions. In contrast, samples with high contributions of cancer-derived DNA should show a drop in coverage, indicative of higher accessibility of the TF binding sites (Fig. 4B).

As pointed out before, exemplary figures of the other report sections, like QC or ichorCNA, can be found in Supplementary Figs. S2–S6). The full example report can be found in our GitHub repository. Finally, we provide supplementary plots showing signals around further transcription factor binding sites (Supplementary Fig. S8), genic features (Supplementary Fig. S9), and around transcription start sites stratified by expression (Supplementary Fig. S10) in comparison to signals shown in Snyder et al. [3].

## Conclusion

Here we propose cfDNA UniFlow, a unified preprocessing pipeline specifically tailored for cfDNA samples. It is an easy-to-use, scalable, and configurable workflow, aiming to set a community standard for enabling accessible and easily sharable future research in the field. In designing our workflow, we aimed at providing a tool that can be used without much computer science background but with the option to be easily extended by experienced users with their own custom modules, allowing its extension from a standard processing workflow to a full-featured analysis pipeline.

## Availability of Source Code and Requirements

Project name: cfDNA UniFlow

Project homepage: https://github.com/kircherlab/cfDNA-UniFlow

Operating system(s): Linux (64-bit)

Programming language: Python

Other requirements: Mamba or Conda

License: MIT

Biotools ID: cfdna_uniflow

RRID: SCR_025600

WorkflowHub SEEK ID: https://doi.org/10.48546/workflowhub.workflow.1091.2 [43]

## Supplementary Material

giae102_GIGA-D-23-00325_Original_Submission

giae102_GIGA-D-23-00325_Revision_1

giae102_GIGA-D-23-00325_Revision_2

giae102_GIGA-D-23-00325_Revision_3

giae102_Response_to_Reviewer_Comments_Original_Submission

giae102_Response_to_Reviewer_Comments_Revision_1

giae102_Response_to_Reviewer_Comments_Revision_2

giae102_Reviewer_1_Report_Original_Submission Wei Zhang -- 12/18/2023 Reviewed

giae102_Reviewer_1_Report_Revision_1 Wei Zhang -- 6/6/2024 Reviewed

giae102_Reviewer_1_Report_Revision_2 Wei Zhang -- 9/3/2024 Reviewed

giae102_Reviewer_2_Report_Original_Submission Zsolt BalÃ¡zs, PhD, MD -- 2/28/2024 Reviewed

giae102_Reviewer_2_Report_Revision_1 Zsolt BalÃ¡zs, PhD, MD -- 6/20/2024 Reviewed

giae102_Supplemental_File

## Data Availability

The data used for generating plots included in this article are available in the European Genome-Phenome Archive [44] with accession EGAD00001010100. The aligned reads from Snyder et al. [3], which were used for Supplementary Figs. 8–10, are deposited in the NCBI Gene Expression Omnibus (GEO) with accession GSE71378 and can be downloaded via the Sequence Read Archive (SRA). Files used for testing cfDNA UniFlow are available on Zenodo [45].

## Additional Files

**Supplementary Note**. A general overview of the workflow including supplementary figures and a quick start guide.

**Supplementary Fig. S1**. A detailed overview of the workflow components.

**Supplementary Fig. S2**. Example section of a QC report.

**Supplementary Fig. S3**. Example report for ichorCNA plot of copy number alterations.

**Supplementary Fig. S4**. Example of a sample’s global GC bias estimate.

**Supplementary Fig. S5**. Example report of regional effects of GC bias correction.

**Supplementary Fig. S6**. Example of a case-control plot.

**Supplementary Fig. S7**. Comparison of 3 fragment-based GC correction methods.

**Supplementary Fig. S8**. Windowed Protection Score around aggregated transcription factor binding sites.

**Supplementary Fig. S9**. Windowed Protection Score around aggregated genic features.

**Supplementary Fig. S10**. Windowed Protection Score around transcription start sites (TSSs) stratified by expression.

**Supplementary Table S1**. Comparison of cfDNA Uniflow and cfDNApipe.

## Abbreviations

BAM: binary alignment map; cfDNA: cell-free DNA; CNA: copy number alteration; ctDNA: circulating tumor DNA; QC: quality control; TF: transcription factor.
