# Supplementary material for: cfDNA UniFlow: a unified preprocessing pipeline for cell-free DNA data from liquid biopsies
Source: Gigascience. 2024 Dec 20;13:giae102. doi: 10.1093/gigascience/giae102 (PMC11659977; doi:10.1093/gigascience/giae102)
Supplement: giae102_Supplemental_File [file giae102_supplemental_file.pdf]

cfDNA UniFlow: A unified preprocessing pipeline for cell-free DNA from liquid biopsies

cfDNA UniFlow is a unified, standardized, and ready-to-use workflow for processing whole genome sequencing (WGS) cfDNA samples from liquid biopsies. It includes essential steps for pre-processing raw cfDNA samples, quality control and reporting. Additionally, several optional utility functions like GC bias correction and estimation of copy number state are included. Finally, we provide specialized methods for extracting coverage derived signals and visualizations comparing cases and controls. Figure S1 gives a detailed overview of the workflow.

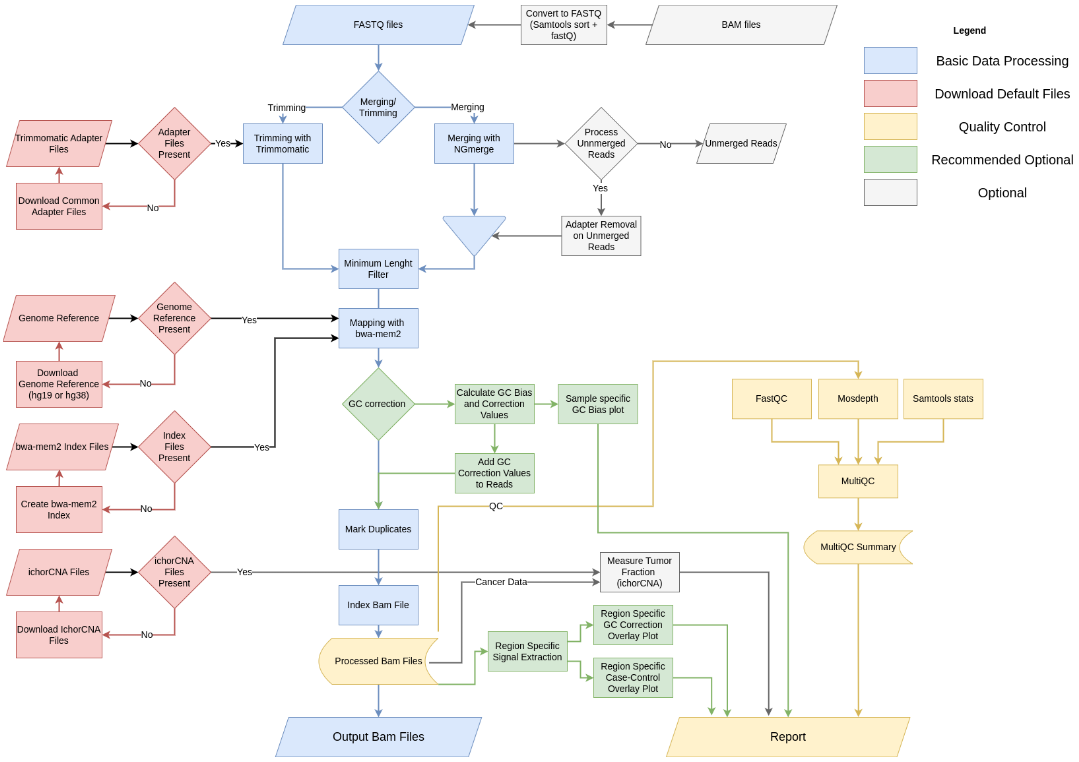

Figure S1: Overview of cfDNA Uniflow. Functionalities are color coded by task. Red boxes represent rules for the automatic download of public resources. Grey boxes are optional steps. Blue boxes contain the core functionalty of cfDNA Uniflow. Green boxes are optional, but highly recommended steps and yellow boxes summarize the Quality Control and reporting steps.

Authors

- Sebastian Röner (@sroener)

Table of Contents

- cfDNA UniFlow: A unified preprocessing pipeline for cell-free DNA from liquid biopsies
    - 1 Dependencies
    - 2 Functional summary
        - 2.1 Raw data processing
        - 2.2 Quality control
        - 2.3 Signal extraction and sequence analysis functionality
        - 2.4 Report
        - 2.5 Comparison of fragment based GC bias correction methods for cfDNA
        - 2.6 Reproducing Signals from Snyder et al. Cell 2016
        - 2.7 Comparison of cfDNA Uniflow with other methods
        - 2.8 Notes on resource requirements
    - 3 Quick-start Guide
        - 3.1 Setup
            - Step 1: Obtain a copy of this workflow
            - Step 2: Install Snakemake
            - Step 3: Activate conda environment
        - 3.2 Integration test
            - Step 1: Download test files
            - Step 2: Check config files
            - Step 3: Executing the workflow
        - 3.3 Running the workflow with the data used in the manuscript
            - Step 1: Download files from EGA
            - Step 2: Check manuscript config files
            - Step 3: Executing the workflow on the manuscript data
            - Step 4: Reproducing figures 2 and 3
        - 3.4 Running the workflow with data from Snyder et al. Cell 2016
            - Step 1: Download and prepare files
            - Step 2: Check config files for Snyder et al. data
            - Step 3: Executing the workflow for Snyder et al. data
            - Step 4: Reproducing Figure S8-10
        - 3.5 Running the workflow with your own data
            - Step 1: Configure samples
            - Step 2: Configure regions
            - Step 3: Configure workflow
            - Step 4: Execute workflow
            - Step 5: Investigate results
    - 4 Project structure
        - 4.1 Config

    - 4.2 Logs
    - 4.3 Resources
    - 4.4 Results
    - 4.5 Workflow
  - 5 Contribution guidelines
  - 6 References

1 Dependencies

To minimize conflicts between packages, all dependencies for the workflow rules are managed via separate conda environments located in `./workflow/envs`. They get automatically installed and used when Snakemake is executed with the `--use-conda` flag. This is the recommended way of executing the workflow.

The only exception is NGmerge, a read merging and adapter removal program, which is included in the GitHub repository due to an outdated bioconda recipe. The NGmerge executable was downloaded and compiled as described in the official documentation of NGmerge v0.3 and is located in the scripts directory (./workflow/scripts/NGmerge). Additionally, we provide an adjusted quality profile for Phred+33 scores of Illumina 1.8+, which ranges from 0 to 41 instead of 0 to 40 in earlier versions. The quality profile file was modified by duplicating the last column and appending it as a new column.

2 Functional summary

2.1 Raw data processing

The core functionality of cfDNA UniFlow is the processing of Whole Genome Sequencing (WGS) data from liquid biopsies. Input data is expected in either FASTQ or BAM format. If a BAM file was provided, it gets converted to FASTQ files using SAMtools. Afterwards, several steps for improving read quality and preparation for mapping are executed, for which two options are provided. Either the recommended merging of reads with NGmerge, which removes adapters and corrects sequencing errors, or trimming of adapters with Trimmomatic. In both cases, results are filtered for a specified minimum read length. Remaining reads are mapped to a reference genome via bwa-mem2. If NGmerge was used for adapter removal, it is possible to include reads in mapping for which only adapters were removed when merging was not possible due to no sufficient overhang between read pairs. The core processing is finalized by marking duplicates and creating a bam index with SAMtools markdup and index. Processed reads are then submitted for Quality Control and optional steps.

2.2 Quality control

In the quality control step, general post-alignment statistics and graphs are calculated for each sample via SAMtools stats and FastQC. Additionally, sample-wide coverage statistics and coverage at different genomic regions are calculated with Mosdepth, a fast BAM/CRAM depth calculation tool for WGS, exome, or targeted sequencing. The QC results are aggregated in HTML report via MultiQC.

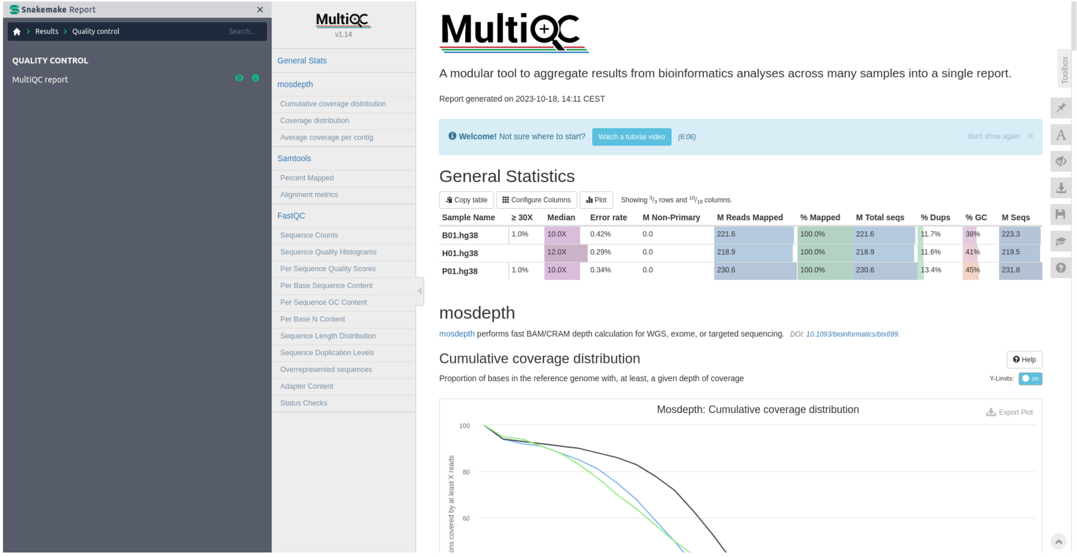

Figure S2: Example section of a QC report. The QC report contains post-alignment statistics from SAMtools stats, FastQC and Mosdepth.

2.3 Signal extraction and sequence analysis functionality

In addition to the preprocessing and quality control functionality, cfDNA UniFlow contains some sequence analysis functions. The first is the widely used tool ichorCNA, which can be used for predicting copy number alteration (CNA) states across the genome. Further, it uses this information for estimating tumor fractions in cfDNA samples. By default, we use the recommended settings for ichorCNA, including profiles provided in the ichorCNA repository. However, it is possible to specify custom profiles and parameters in the configuration file.

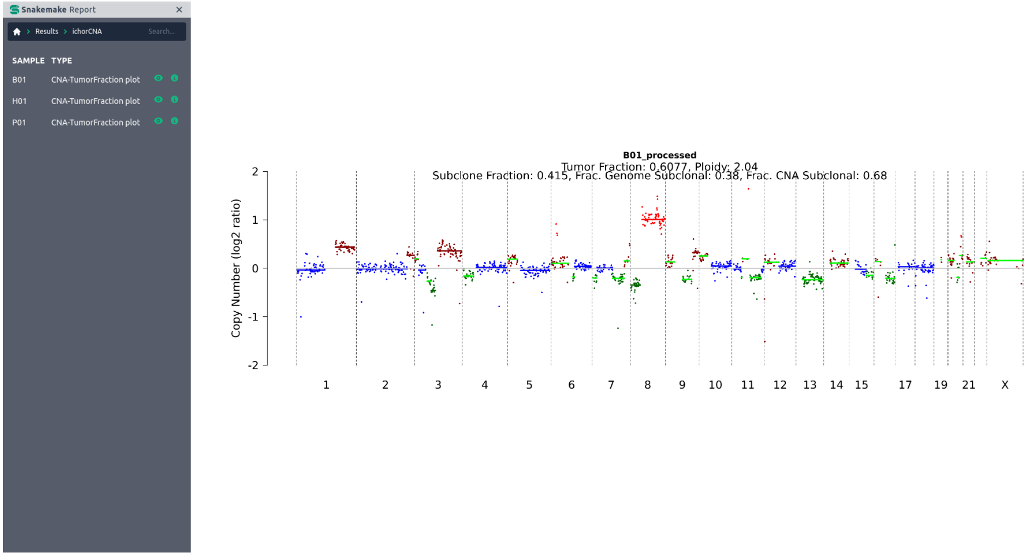

Figure S3: ichorCNA plot of copy number alterations. The plot shows genome wide annotation of estimated copy number alterations, tumor fraction and other parameters used by ichorCNA. This example was generated using a breast cancer sample with an average GC content of 38%.

The second utility function is our inhouse GC bias estimation method. It can not only be used for estimating fragment length and GC-content dependent technical biases, but also includes the option of attaching correction values to the reads. These can be used downstream for a wide variety of signal extraction methods, while preserving the original read coverage patterns. Additionally, it is possible to include a visualization of the estimated biases in the report.

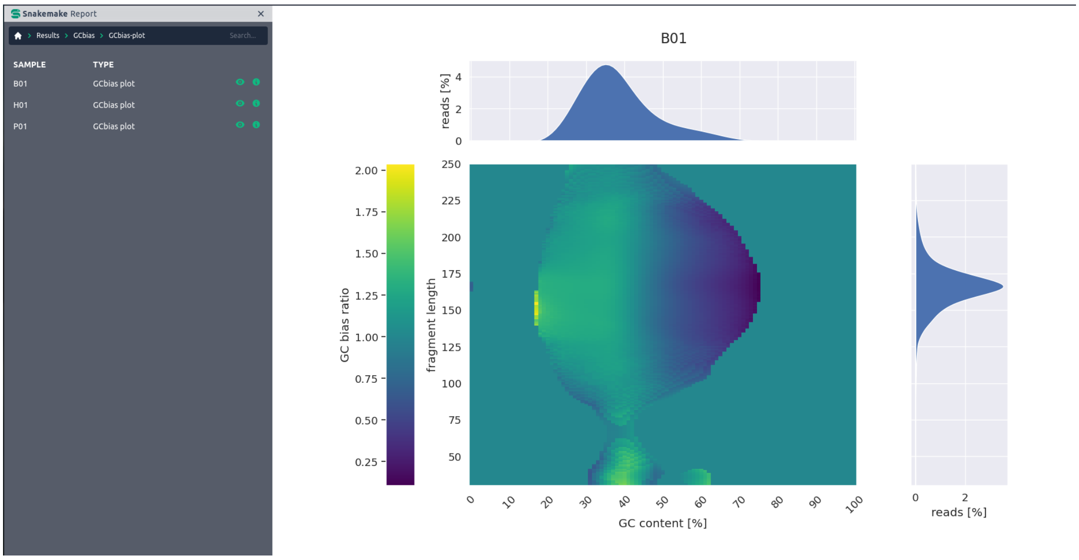

Figure S4: Global GC bias estimate. The global GC bias was estimated using ratio between observed and expected fragments stratified by GC content [%] and fragment length [bp].This example was generated using a breast cancer sample with an average GC content of 38%.

Building on the GC bias estimation, we provide a method for extracting coverage derived signals around user defined regions. The resulting signals can be visualized for comparing biased vs corrected states and for comparing cases against controls.

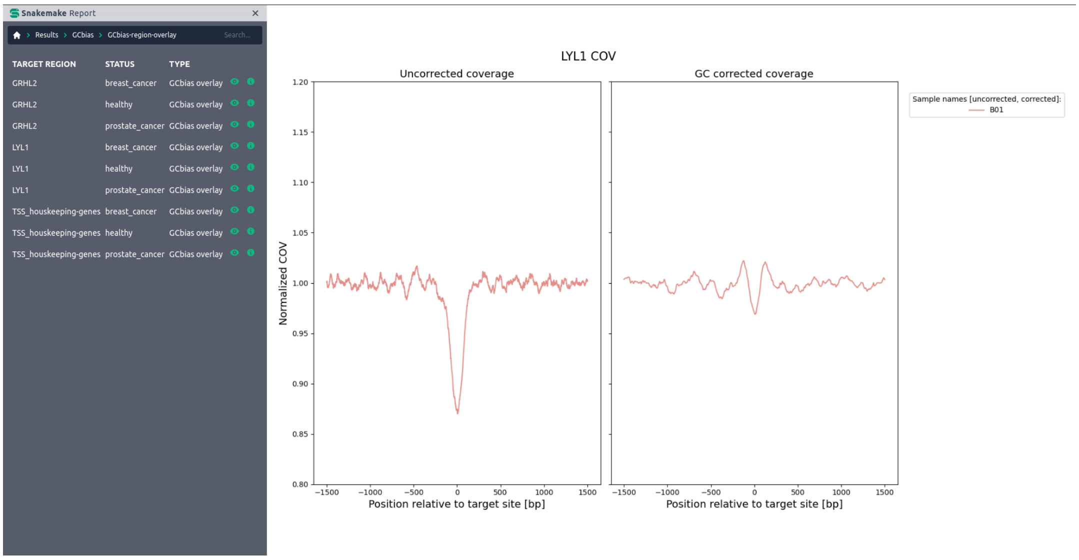

Figure S5: Regional effects of GC bias correction. Composite coverage signal of 10000 LYL1 binding sites before and after GC bias correction. Accessibility of the binding sites, indicated by lower coveragem would have been overestimated before correction. This example was generated using a breast cancer sample with an average GC content of 38%.

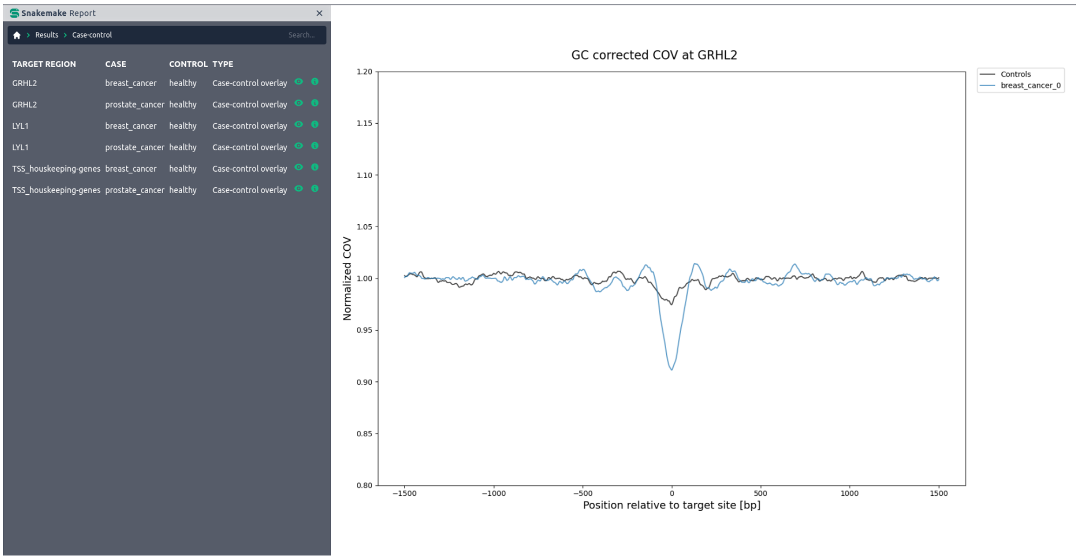

Figure S6: Case-control plot. GC corrected composite coverage signal of 10000 GRHL2 binding sites in a healthy control and a breast cancer sample. Lower central coverage in the cancer sample is consistent with increased GRHL2 activity in many cancer types.

2.4 Report

Finally, all results and summary statistics for the specified samples are aggregated in one report, making a wide variety of information easily accessible. The report file is generated using Snakemake's report feature. After the workflow finished, the report can be generated by executing `Snakemake –configfile <CONFIGFILE> --snakefile <SNAKEFILE> --report <REPORTNAME>.html`. For better report performance, it is recommended to use `--report <REPORTNAME>.zip`, which creates a zipped directory structure with the needed information instead of saving it in the HTML file itself. More information on reporting can be found in the official Snakemake documentation.

Note: An example report can be found here

2.5 Comparison of fragment based GC bias correction methods for cfDNA

The figure below shows a comparison of fragment based GC bias correction methods for cfDNA. We compared our inhouse method (ID: cfDNA Uniflow) with the methods from GCparagon [1] and scores extracted from the Griffin workflow [2]. Expected and measured fragment distributions were sampled from 1e5 random regions of 1 kilobase (kb) width, covering 1e8 base pair (bp) in total. The expected fragment distribution is based on all possible fragments of size 20 to 550 bp and their respective GC content based on the human genome reference hg38. The measured fragment distribution is based on the actual fragments in the cfDNA sample.

A)

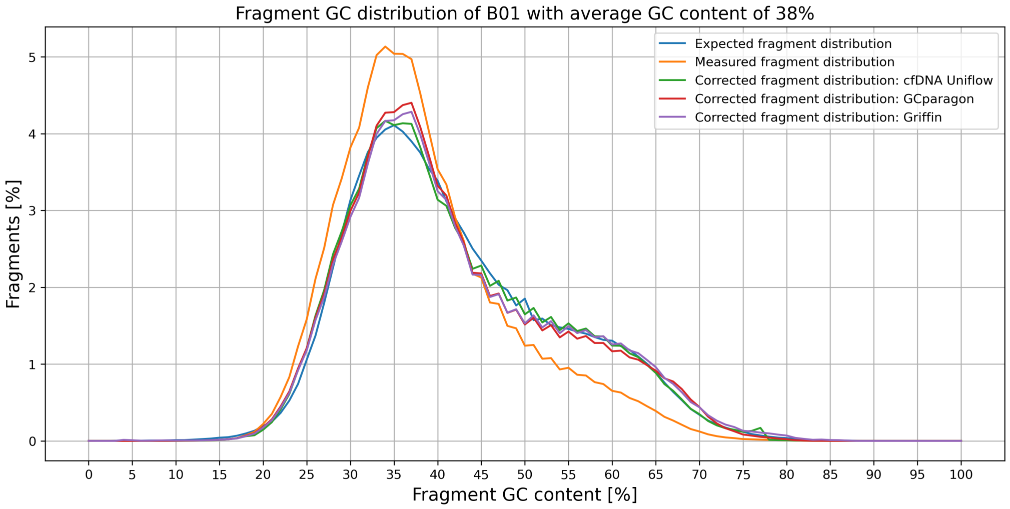
Fragment GC distribution of B01 with average GC content of 38%
Expected fragment distribution
Measured fragment distribution
Corrected fragment distribution: cfDNA Uniflow
Corrected fragment distribution: GCparagon
Corrected fragment distribution: Griffin
Fragments [%]
Fragment GC content [%]

B)

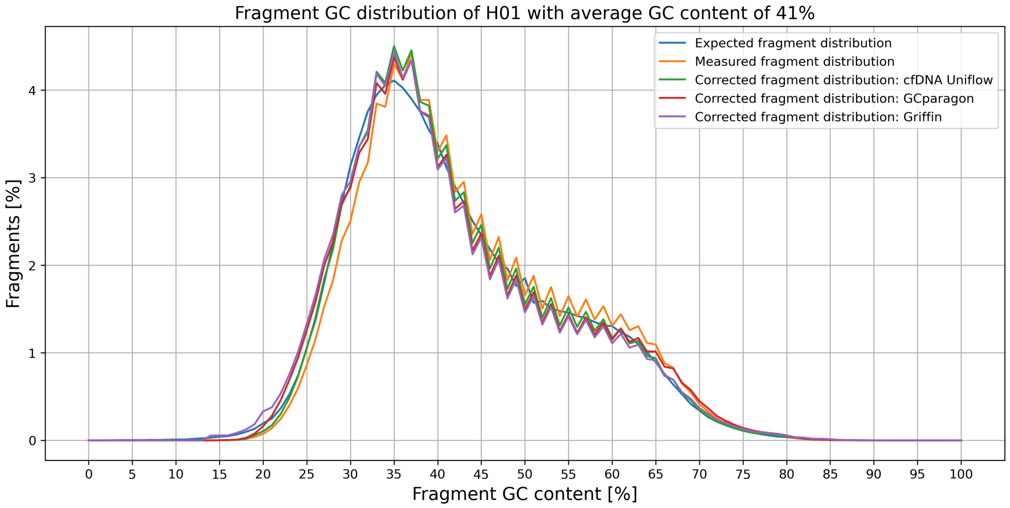
Fragment GC distribution of H01 with average GC content of 41%
Expected fragment distribution
Measured fragment distribution
Corrected fragment distribution: cfDNA Uniflow
Corrected fragment distribution: GCparagon
Corrected fragment distribution: Griffin
Fragments [%]
Fragment GC content [%]

C)

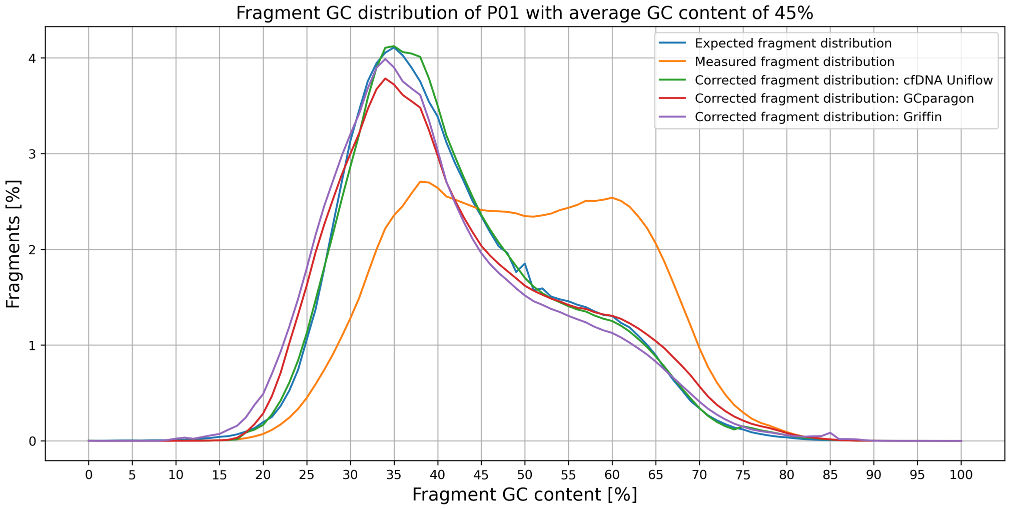
Fragment GC distribution of P01 with average GC content of 45%
Expected fragment distribution
Measured fragment distribution
Corrected fragment distribution: cfDNA Uniflow
Corrected fragment distribution: GCparagon
Corrected fragment distribution: Griffin
Fragments [%]
Fragment GC content [%]

Figure S7: Comparison of three fragment-based GC bias correction methods for cfDNA. The expected fragment distribution is based on all possible fragments of size 20 to 550 bp and their respective GC content based on the human genome reference hg38. The measured fragment distribution is based on the actual fragments in the cfDNA sample. Both distributions were sampled from 1e5 random regions of 1 kilobase (kb) width, covering 1e8 base pair (bp) in total. Corrected fragment distributions were calculated by multiplying the fragment counts with GC correction factors of the respective methods. Subfigure A) show the effects for the tested sample B01 (38% average GC), B) for sample H01 (41% average GC) and C) for sample P01 (47% average GC).

2.6 Reproducing Signals from Snyder et al. Cell 2016

In the following section, plots are shown that were generated using the cfDNA UniFlow workflow and the data from the Snyder et al. Cell 2016 paper. The figures demonstrate that the workflow, even though a different processing, additional bias correction steps and refinement of raw signals were used, can reproduce the signals from the original publication. Figure S8 shows signals around transcription factor binding sites (TFBS) of CTCF, MAFK, and ETS, as shown in Snyder et al. [3]. Additionally, Figure S9 shows the signals around genomic features like Transcription Start Sites (TSSs), Transcription End Sites (TESs), splice sites, and start- and stop codons. Finally, Figure S10 shows signals around TSSs stratified by expression levels, as shown in Snyder et al. [3]. A report containing further information on the processed files can be found in the supplement directory and needs to be unzipped before viewing.

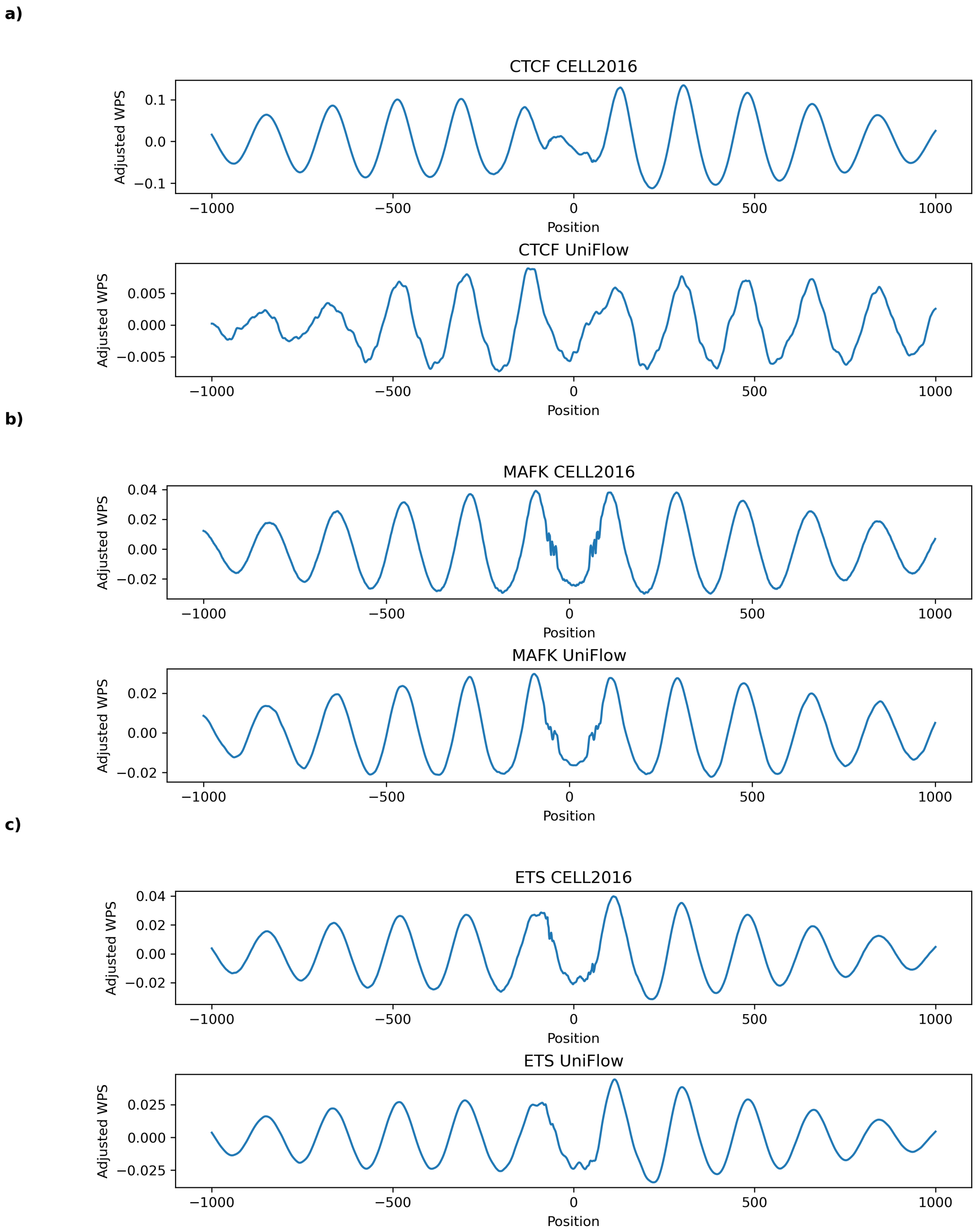

Figure S8: Windowed Protection Score around aggregated transcription factor binding sites. Each panel shows two plots. One showing signals extracted from data as shown in Snyder et al. using the original processing steps and one showing signals extracted using cfDNA UniFlow. Panel a) shows signals around CTCF binding sites, panel b) shows signals around MAFK binding sites and panel c) shows signals around ETS binding sites. Differences in the signals are due to different processing steps of raw reads and additional bias correction in cfDNA UniFlow.

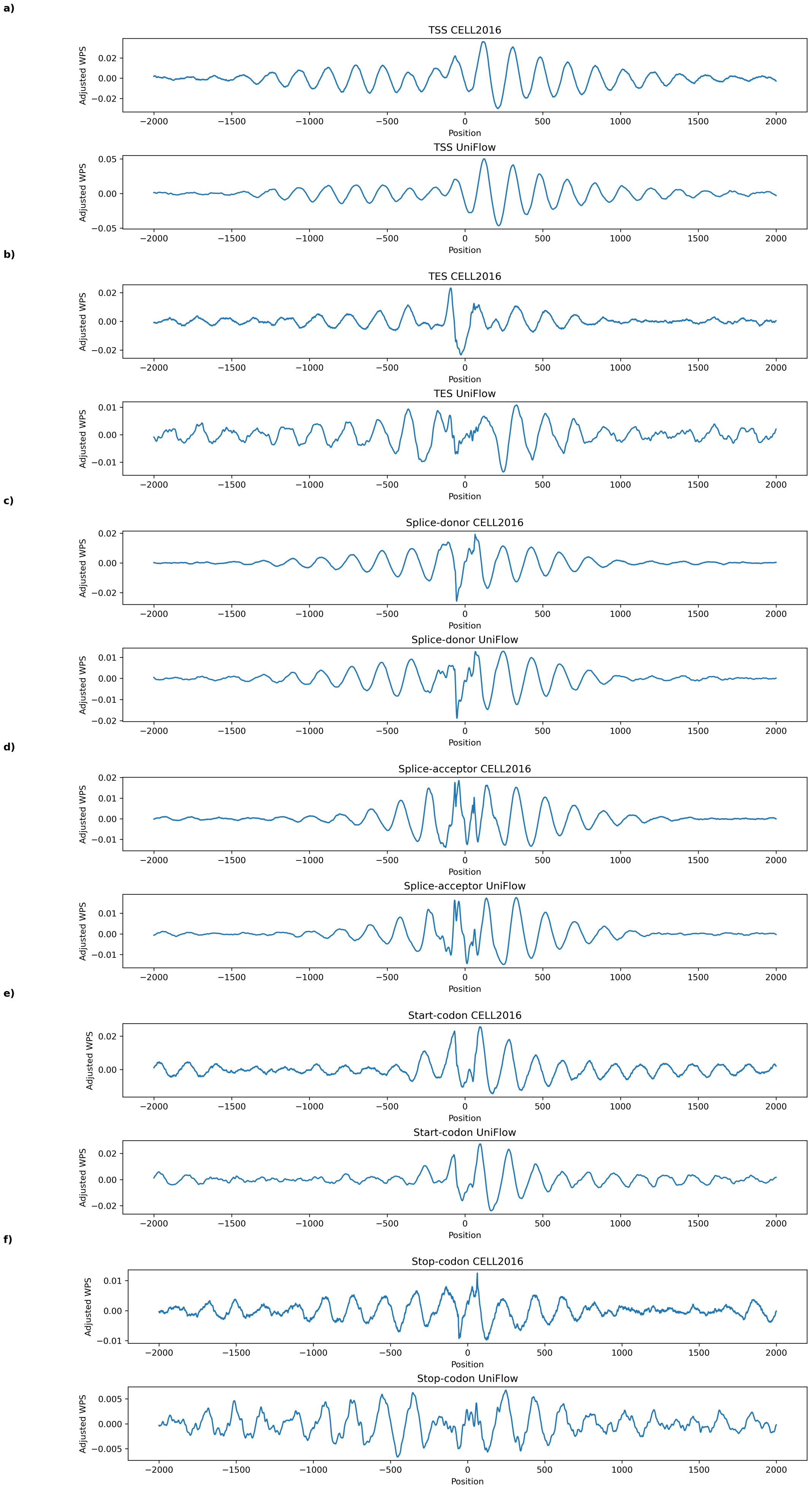
a)
TSS CELL2016
Adjusted WPS
Position
TSS UniFlow
Adjusted WPS
Position
b)
TES CELL2016
Adjusted WPS
Position
TES UniFlow
Adjusted WPS
Position
c)
Splice-donor CELL2016
Adjusted WPS
Position
Splice-donor UniFlow
Adjusted WPS
Position
d)
Splice-acceptor CELL2016
Adjusted WPS
Position
Splice-acceptor UniFlow
Adjusted WPS
Position
e)
Start-codon CELL2016
Adjusted WPS
Position
Start-codon UniFlow
Adjusted WPS
Position
f)
Stop-codon CELL2016
Adjusted WPS
Position
Stop-codon UniFlow
Adjusted WPS
Position

Figure S9: Windowed Protection Score around aggregated genic features. Each panel shows two plots. One showing signals extracted from data as shown in Snyder et al. using the original processing steps and one showing signals extracted using cfDNA UniFlow. Panel a) shows signals around TSSs, panel b) shows signals around TESs, panel c) shows signals around splice sites, panel d) shows signals around start codons and panel e) shows signals around stop codons. Differences in the signals are due to different processing steps of raw reads and additional bias correction in cfDNA UniFlow.

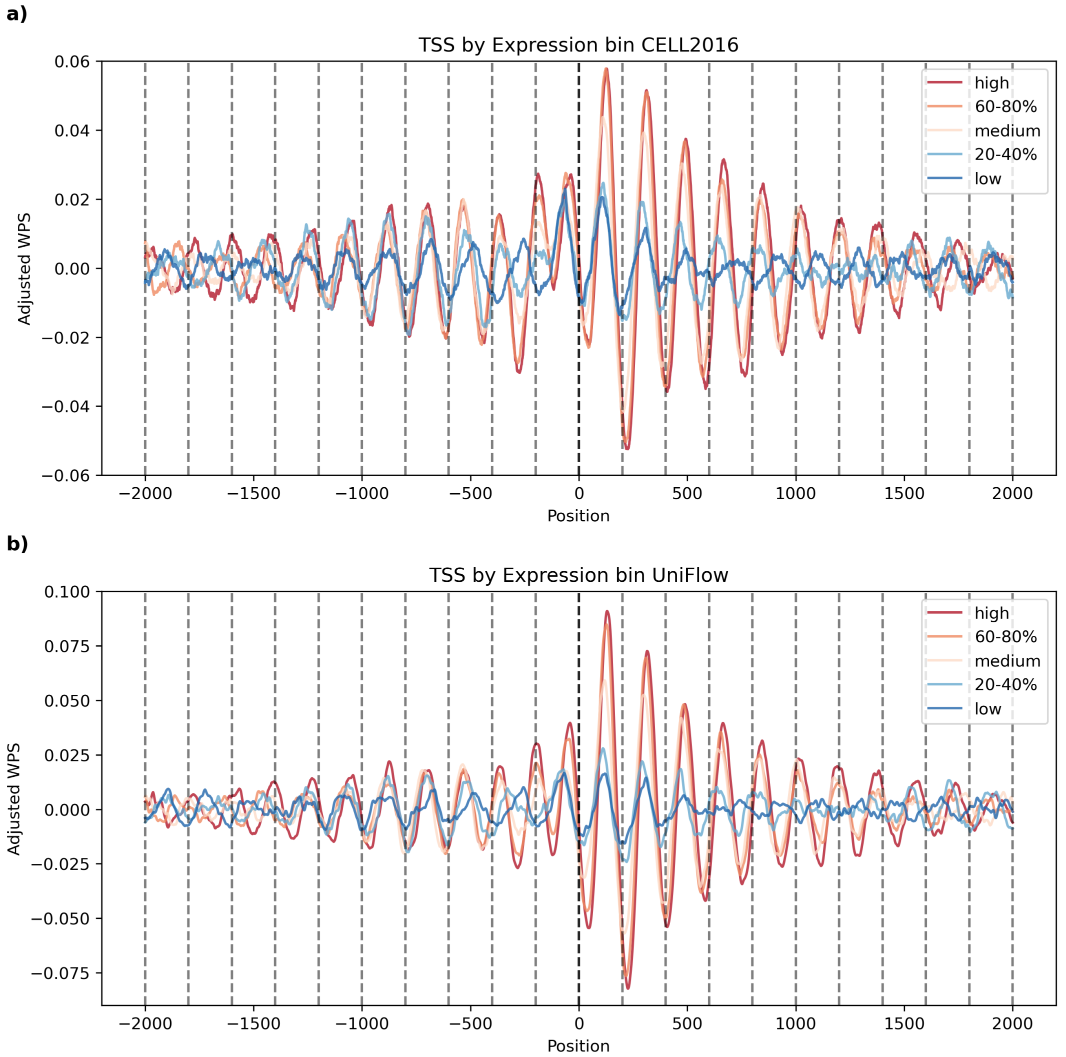

Figure S10: Windowed Protection Score around TSSs stratified by expression. Panel a) shows signals extracted from data as shown in Snyder et al. and panel b) shows signals extracted using cfDNA UniFlow. In both cases, signals are stratified by expression levels, showing stronger phasing around highly expressed genes.

2.7 Comparison of cfDNA Uniflow with other methods

The table S1 shows a comparison of cfDNA Uniflow with the cfDNApipe workflow. Both workflows are designed for the preprocessing of cfDNA samples from liquid biopsies, but follow different design principles.

Table S1: Comparison cfDNA Uniflow vs cfDNApipe.

Characteristic	cfDNA Uniflow	cfDNApipe
**Workflow characteristics**		
package management	conda	conda
language	Snakemake	Python
modularity	yes	no
multiprocessing support	yes	yes
configurability	config files	custom python scripts
scalability	single machine to compute clusters	single machine
**Core functionality**		
Adapter removal	Ngmerge or Trimmomatic	adapterremoval
Mapping	bwa-mem2	Bowtie2/Bismark
Length filtering	Yes	-
Sequencing error correction	Ngmerge	-
GC correction	Fragment level	Bin or arm level (CNV only)
Quality Control	FastQC, Samtools stats, Mosdepth	FastQC, Qualimap
Reporting	Snakemake + integrated MultiQC report	Custom html report
**Analyses**		
CNV analysis	IchorCNA	Jiang P. et al. (2015)
Tumor fraction estimation	IchorCNA	-
Methylation analysis	-	case-control + tissue deconvolution
Fragmentomic analysis	yes	yes
Nucleosome analysis	yes	yes
SNV/InDel analysis	possible extension	yes (calling only)
Virus detection	possible extension	yes

2.8 Notes on resource requirements

- The index creation of bwa-mem2 is resource intensive:

```
# Indexing the reference sequence (Requires 28N GB memory where N is
the size of the reference sequence).
./bwa-mem2 index [-p prefix] <in.fasta>
Where
<in.fasta> is the path to reference sequence FASTA file and
<prefix> is the prefix of the names of the files that store the
resultant index. Default is in.FASTA.
```

More information can be found in the documentation or this issue.

- bwa-mem2 mem uses around 4GB memory per thread.

3 Quick-start Guide

3.1 Setup

The goal of cfDNA Uniflow is to provide essential and standardized preprocessing steps in a reproducible and scalable way, with the option to include additional steps as needed. Therefore, we encourage users to use this workflow as a template and build their own analysis on top of it. The following steps guide you through the setup of the workflow.

Note 1: If you use this workflow in a paper, don't forget to give credits to the authors by citing the URL of this (original) repository.

Note 2: If you want to contribute to the development of cfDNA UniFlow, please follow the guidelines in section 4.

Step 1: Obtain a copy of this workflow

1. Create a new GitHub repository using this workflow as a template.
2. Clone the newly created repository to the local system, into the place where the data analysis will be performed.

Step 2: Install Snakemake

After successful installation, set up an environment for Snakemake. This can be done by executing the following command:

```
conda create -c bioconda -c conda-forge -n snakemake snakemake
```

The environment can be activated via the `conda activate snakemake` command.

For installation details, see the instructions in the Snakemake documentation.

Installation instructions for conda can be found in the official conda documentation.

Step 3: Activate conda environment

Activate the conda environment:

```
conda activate snakemake
```

3.2 Integration test

Our goal in developing cfDNA UniFlow is to provide a scalable, configurable, and easy-to-use workflow specifically tailored towards the processing of cfDNA samples. Users only need to provide sequencing information in FASTQ or BAM format and optionally modify the configuration file to their needs. Here we provide an example with a small input file for testing the workflows functionality.

Note: As this is an integration test, the generated data is not intended to generate meaningful results. This test is only meant to check if the workflow is running without errors.

Step 1: Download test files

First, the expected directory for the test files needs to be created:

```
mkdir -p resources/testsample/
```

Afterwards, the test-files located on this webserver need to be downloaded:

```
wget -r -nd --directory-prefix=resources/testsample/ --reject="index.html*" --no-parent --tries=inf --continue https://kircherlab.bihealth.org/download/cfDNA-testSample/
```

Alternatively, the test-files can be downloaded manually from Zenodo and be placed in the `resources/testsample/` directory.

Step 2: Check config files

There are five files that are used in this example:

- config/test-config.yaml
- config/test-config_fastq.yaml
- config/test-samples.tsv
- config/test-samples_fastq.tsv
- config/test-regions.tsv

Note: These files are configured for a quick integration test and shouldn't be edited.

Step 3: Executing the workflow

The last step is executing the Workflow from the root directory of the cloned repository.

Test the configuration with a dry-run using the following command:

```
snakemake --use-conda --configfile config/test-config.yaml -n
```

or

```
snakemake --use-conda --configfile config/test-config_fastq.yaml -n
```

if the test should be executed using FASTQ files.

The workflow is executed locally with $N cores via:

```
snakemake --use-conda --configfile config/test-config.yaml --cores $N
```

For cluster execution, read the guidelines in the Snakemake documentation.

Note: The only difference between the two configs is the input file format (BAM or FASTQ). If a BAM file is used, the workflow will convert it to FASTQ files before processing. Otherwise, the FASTQ files are used directly.

3.3 Running the workflow with the data used in the manuscript

Step 1: Download files from EGA

The data used in the manuscript is available on the European Genome-Phenome Archive (EGA) under the accession number EGAD00001010100. For the download, you need to have an EGA account and request access via the EGA website. Afterwards, the data can be downloaded using the EGA download client.

After downloading the data, the files need to be placed in the directory `resources/manuscript_samples/` relative to the repository root. If the directory does not exist, create it with the following command:

```
mkdir -p resources/manuscript_samples/
```

Step 2: Check manuscript config files

We provide a configuration file for the manuscript data in the `supplement/manuscrip_analysis` directory. The directory contains the following files:

- manuscript_config.yaml
- manuscript_samples.tsv
- cfDNA_Uniflow_figure2-3.ipynb

Note: Please check the paths/file names in the `manuscript_samples.tsv` file and adjust them if necessary.

Step 3: Executing the workflow on the manuscript data

This step is similar to the integration test, but with the manuscript data.

Test the configuration with a dry-run:

```
snakemake --use-conda --configfile supplement/manuscrip_analysis/manuscript_config.yaml -n
```

The workflow is executed locally with $N cores via:

```
snakemake --use-conda --configfile supplement/manuscrip_analysis/manuscript_config.yaml --cores $N
```

If possible, we highly encourage the execution of the workflow in a cluster environment. The detailed configuration depends on the available infrastructure. More information can be found in the respective subsection of the Snakemake documentation.

Step 4: Reproducing figures 2 and 3

To reproduce the figures 2 and 3 from the manuscript, the Jupyter notebook `cfDNA_Uniflow_figure2-3.ipynb` located in the `supplement/manuscript_analysis` directory needs to be executed. The notebook contains the code for generating the figures and is based on the results generated by the workflow.

3.4 Running the workflow with data from Snyder et al. Cell 2016

Step 1: Download and prepare files

The aligned reads from the Snyder et al. Cell 2016 [3] paper are deposited in the NCBI Gene Expression Omnibus (GEO) with accession GSE71378 and can be downloaded via SRA. A copy of the original BAM files is also provided on the Kircherlab webserver for convenience.

The files can be downloaded with the following commands:

```
# Create the expected directory
mkdir -p resources/snyder_et_al_2016/
# Download the files
wget -r -nd --directory-prefix=resources/snyder_et_al_2016/ --reject="index.html*" --no-parent --tries=inf --continue https://kircherlab.bihealth.org/download/cfDNA/
```

To reproduce the plots described in section 2.6, the sample `CH01.bam` needs to be created by merging the samples `BH01.bam`, `IH01.bam` and `IH02.bam`:

```
samtools merge -o resources/snyder_et_al_2016/CH01.bam resources/snyder_et_al_2016/BH01.bam resources/snyder_et_al_2016/IH01.bam resources/snyder_et_al_2016/IH02.bam
```

Afterwards, the respective entry has to be added to the `CELL2016_samples.tsv` file.

Step 2: Check config files for Snyder et al. data

Configuration files for the Snyder et al. data are provided in the `supplement/Snyder-et-al_Cell2016_analysis` directory. The directory contains the following files:

- CELL2016_config.yaml
- CELL2016_samples.tsv
- CELL2016_regions.tsv
- CELL2016_region_files
- Cell2016_data
- Snyder-et-al_Cell2016_report.zip
- Reproduce_cell2016_plots.ipynb

Note: Please check the paths/file names in the `CELL2016_samples.tsv` file and adjust them if necessary.

Step 3: Executing the workflow for Snyder et al. data

Test the configuration with a dry-run:

```
snakemake --use-conda --configfile supplement/Snyder-et-al_Cell2016_analysis/CELL2016_config.yaml -n
```

The workflow is executed locally with $N cores via:

```
snakemake --use-conda --configfile supplement/Snyder-et-al_Cell2016_analysis/CELL2016_config.yaml --cores $N
```

If possible, we highly encourage the execution of the workflow in a cluster environment. The detailed configuration depends on the available infrastructure. More information can be found in the respective subsection of the Snakemake documentation.

Step 4: Reproducing Figure S8-10

To reproduce the figures S8-10, the workflow in step 3 needs to be executed and finished. Afterwards, plots can be generated using the provided Jupyter notebook `cfDNA_Uniflow_figure2-3.ipynb` located in the `supplement/Snyder-et-al_Cell2016_analysis` directory.

3.5 Running the workflow with your own data

After running the integration test, the workflow with your own data. For this, you need to modify the configuration files and provide your own data. All three configuration files (`samples.tsv`, `regions.tsv`, `config.yaml`) should be located in the `config/` directory and will be automatically validated before workflow execution. All other default files are either included, or will be downloaded and generated automatically.

Note: Names of the configuration files are arbitrary and can be changed according to your needs, but have to be referenced correctly in the Snakemake command and `config.yaml`.

Step 1: Configure samples

The `samples.tsv` file should contain the relevant information for each sample in an experiment. It should contain the following columns:

```
# Identifier of the cohort/experiment
ID: string
# Sample name/identifier
sample: string
# Path to the BAM file (for reprocessing); insert "-" if FASTQ files
are used
bam: string
# Path to the R1 FASTQ file; insert "-" if bam is used
fq1: string
# Path to the R2 FASTQ file; insert "-" if bam is used
fq2: string
# Name of the target genome build (hg19 or hg38)
genome_build: string
# Name of the library prep (e.g. ThruPLEX DNA-seq)
library_name: string
# Platform used for sequencing (e.g. Illumina NextSeq 500)
platform: string
# Status of the sample (e.g. Case/Control or Healthy/Cancer)
status: string
# Additional information that should be included in read group ID
(e.g. condition)
info: string
```

If FASTQ files are used, either single-end or paired-end sequencing can be specified, depending on how many FASTQ files are provided. If BAM files are used, the FASTQ columns should be filled with "-" and vice versa. Examples for either BAM or FASTQ based sample configuration can be found in the `config/test-samples.tsv` file, or `config/test-samples_fastq.tsv` file, respectively.

Step 2: Configure regions

The `regions.tsv` file contains names and paths to the bed files for regions of interest. It should contain the following columns:

```
# region name/identifier
target: string
# path to the bed files for regions of interest
path: string
```

Step 3: Configure workflow

The `config.yaml` is the main configuration file for the workflow. It contains options for the workflow execution and paths to the configuration files. We provide sensible defaults and document the purpose of most options via comments in the `config.yaml`, but the user can adjust them according to their needs.

In the following examples, we cover the most important options:

```
samples: "config/test-samples.tsv"
regions: "config/test-regions.tsv"
control_name: "healthy"
```

The first section of the config file should be used to specify the paths to the samples and regions files. The `control_name` option is used to specify the name of the control samples in the samples file, which will be used for the case-control plot.

```
### genome build specific options ###

hg19:
  reference: "resources/reference/hg19.fa"
  2bit_ref: "resources/reference/hg19.2bit"
  repeatmasker:
    "resources/blacklists/repeatmasker.hg19.merged.bed.gz"
  effectiveGenomeSize: 2864785220

hg38:
  reference: "resources/reference/hg38.fa"
  2bit_ref: "resources/reference/hg38.2bit"
  repeatmasker:
    "resources/blacklists/repeatmasker.hg38.merged.bed.gz"
  effectiveGenomeSize: 2913022398
```

The second section of the config file contains genome build specific options. The paths to the reference genome, 2bit reference, repeatmasker blacklist, and effective genome size are specified for both hg19 and hg38. If defaults are used, the workflow will download the necessary files automatically. If these files are already available, the paths should be adjusted accordingly.

```
### global variables ###

TMPDIR: "$TMPDIR" # path to directory for writing TMP files

SEED: 42 # seed for increased reproducibility. Mainly used in GCbias
estimation

### Utility options ###

utility:
  GCbias-plot: True
  GCbias-correction: True
  ichorCNA: True
  case-control-plot: True

### trimming ###

PE_trimming_algorithm: "NGmerge" #can be either NGmerge or
trimmomatic
```

The third section of the config file contains global variables and configuration of optional steps in the workflow. The `TMPDIR` variable specifies the path to the directory for writing temporary files. The `SEED` variable is used for increased reproducibility and is mainly used in the GC bias estimation. The `utility` section is used to enable or disable optional steps in the workflow using either `True` or `False`. Finally, `PE_trimming_algorithm` variable specifies the algorithm used for trimming paired-end reads. It can be either `NGmerge` or `trimmomatic`. All subsequent options are specific

options rules/programs in the workflow and do not need to be adjusted for most use cases.

Note: The configuration file is validated before the workflow execution. If essential information is missing or false values are provided, the workflow will report an error and not be executed.

Step 4: Execute workflow

Activate the conda environment:

```
conda activate snakemake
```

Test your configuration by performing a dry-run with `<CONFIGFILE>` representing the path to your modified config file via:

```
snakemake --use-conda --configfile <CONFIGFILE> -n
```

Execute the workflow locally using `$N` cores:

```
snakemake --use-conda --configfile <CONFIGFILE> --cores $N
```

See the Snakemake documentation on workflow execution and execution in cluster environments for further details.

Step 5: Investigate results

After successful execution, you can create a self-contained interactive HTML report with all results via:

```
snakemake --configfile <CONFIGFILE> --report report.html
```

This report can, e.g., be forwarded to your collaborators.

A functional description of reporting can be found in section 2.4.

An example report in zip format can be found in the supplement directory. For viewing, the zip file needs to be extracted.

4 Project structure

The workflow produces a typical project structure:

```
.
├── config
├── logs
├── resources
├── results
└── workflow
```

Each of the listed directories has a specific purpose, as described in the respective subsections:

4.1 Config

The `config` directory typically contains the configuration files for the workflow. The files are used to specify the samples, regions of interest, and workflow settings. Each of these can be reused for multiple runs if desired.

4.2 Logs

The `logs` directory contains log files generated by workflow rules during execution, documenting the progress and potential errors. Log files are placed in subdirectories according to ID and rule names.

```
.
├── logs
|   ├── <EXPERIMENT ID1>
|   |   ├── <RULE NAME1>
|   |   ├── <RULE NAME2>
|   |   ├── ...
```

4.3 Resources

The `resources` directory contains all files that are needed for the workflow, but not specific for an experiment/run. Most of the necessary files are downloaded automatically, including reference genomes, adapter sequences, and blacklists. The directory is structured as follows:

```
.
├── resources
│   ├── adapter
│   ├── blacklists
│   ├── ichorCNA
│   ├── reference
│   ├── testregions
│   └── testsample
```

4.4 Results

The `results` directory contains all results generated by the workflow. The directory is structured as follows:

```
.
├── results
│   ├── <Experiment ID1>
│   │   ├── corrected_reads
│   │   ├── fastq
│   │   ├── GCBias
│   │   │   ├── bias_table
│   │   │   └── plots
│   │   ├── GCBias_background
│   │   ├── ichorCNA
│   │   │   ├── <SAMPLE ID1>_processed_<GENOME_BUILD>
│   │   │   │   └── <SAMPLE ID1>_processed
│   │   │   ├── chroms
│   │   │   └── readcounts
│   │   ├── mapped_reads
│   │   ├── NGmerge
│   │   │   ├── merged
│   │   │   └── nonmerged
│   │   ├── qc
│   │   │   ├── fastqc
│   │   │   ├── mosdepth
│   │   │   ├── multiqc_data
│   │   │   └── samtools-stats
│   │   ├── signals
│   │   │   ├── case-control-plots
│   │   │   ├── controls
│   │   │   ├── GCcorrection-plots
│   │   │   ├── signal-corrected
│   │   │   └── signal-uncorrected
│   │   └── trimmed
│   │       └── trimmomatic
│   ├── <Experiment ID2>
│   │   ├── ...
```

The results are structured by experiment ID and contain subdirectories for each step of the workflow. To save disk space, intermediate results (e.g. trimmed reads) are

removed after they are no longer needed by downstream tasks. If all intermediate results should be kept, the `--notemp` flag can be used in the Snakemake command.

Note: The easiest way to aggregate results is to use the integrated report feature.

4.5 Workflow

The `workflow` directory contains all scripts, environments, schemas and rules for the workflow, with the Snakefile as main entry point. The directory is structured as follows:

```
.
└── workflow
    ├── envs
    ├── report
    ├── rules
    ├── schemas
    ├── scripts
    └── Snakefile
```

The `envs` subdirectory contains all conda environments used in the workflow. If a new environment is needed, it should be created here.

The `report` subdirectory contains the templates for the report generation. The templates are written in Jinja2 and can be customized according to the user's needs.

The `rules` subdirectory contains all rules for the workflow. Rules are grouped by functionality and are written in the Snakemake language. The `common.smk` file contains utility functions used throughout the workflow. If new rules are needed, they should be added here.

The `schemas` subdirectory contains the JSON schemas used for validating the configuration files.

The `scripts` subdirectory contains all scripts used in the workflow which are not installed via conda environments. If new scripts are needed, or existing scripts need to be modified, they should be added or changed here.

The `Snakefile` is the main entry point for the workflow. It contains target rule for the workflow execution and imports all rules from the `rules` directory.

5 Contribution guidelines

In case you want to contribute to the development of cfDNA UniFlow and its core functionalities, please follow these steps:

1. Fork the original repo to a personal or lab account.
2. Clone the fork to your local system, to a different place than where you ran your analysis.
3. Modify the code according to the intended changes.

4. Commit and push your changes to your fork.
5. Create a pull request against the original repository.

6 References

[1] Spiegl B, Kapidzic F, Röner S, Kircher M, Speicher MR. GCparagon: evaluating and correcting GC biases in cell-free DNA at the fragment level. NAR Genomics Bioinforma. 2023; doi: 10.1093/nargab/lqad102.

[2] Doebley A-L, Ko M, Liao H, Cruikshank AE, Santos K, Kikawa C, et al. A framework for clinical cancer subtyping from nucleosome profiling of cell-free DNA. Nat Commun. 2022; doi: 10.1038/s41467-022-35076-w.

[3] Snyder MW, Kircher M, Hill AJ, Daza RM, Shendure J. Cell-free DNA Comprises an In Vivo Nucleosome Footprint that Informs Its Tissues-Of-Origin. Cell. 2016; doi: 10.1016/j.cell.2015.11.050.
