# Supplementary material for: cfDNA UniFlow: a unified preprocessing pipeline for cell-free DNA data from liquid biopsies
Source: Gigascience. 2024 Dec 20;13:giae102. doi: 10.1093/gigascience/giae102 (PMC11659977; doi:10.1093/gigascience/giae102)
Supplement: giae102_Response_to_Reviewer_Comments_Original_Submission [file giae102_response_to_reviewer_comments_original_submission.pdf]

Author's Response To Reviewer Comments

Gigascience Technical Note: cfDNA UniFlow (GIGA-D-23-00325)

Dear Ms Hongling Zhou,

We thank you and the reviewers for the valuable input.

Please find attached a revised version of our manuscript and detailed answers to the reviewers' questions (as a formatted PDF as Supplemental Material). We would like to highlight the following (relatively minor) changes:

- We added “data” to the title: “cfDNA UniFlow: A unified preprocessing pipeline for cell-free DNA data from liquid biopsies”.
- We updated Figure 1 to highlight implemented versus potential third-party analyses and changed “Utility functions” to “Sequence analysis” to clarify the application of tools listed in this category.
- We changed a section in the “Preprocessing” paragraph to clarify read trimming options.
- We integrated requested minor changes and fixed typos.
- We added a more detailed description on how to reproduce our results in our GitHub repository, providing documentation, configuration files and additional code.
- We added a plot comparing our GC correction method to two other methods, illustrating the effects in a new supplemental figure (Figure S7)
- We updated the supplement document accordingly

Thank you again for considering our manuscript for publication at Gigascience and we look forward to hearing from you.

With best regards (on behalf of the authors),

Martin Kircher
