# Supplementary material for: cfDNA UniFlow: a unified preprocessing pipeline for cell-free DNA data from liquid biopsies
Source: Gigascience. 2024 Dec 20;13:giae102. doi: 10.1093/gigascience/giae102 (PMC11659977; doi:10.1093/gigascience/giae102)
Supplement: giae102_Response_to_Reviewer_Comments_Revision_1 [file giae102_response_to_reviewer_comments_revision_1.pdf]

Author's Response To Reviewer Comments

A cover letter and a response to the reviewer comments has been attached as Supplementary Files to the submission.
