# Supplementary material for: cfDNA UniFlow: a unified preprocessing pipeline for cell-free DNA data from liquid biopsies
Source: Gigascience. 2024 Dec 20;13:giae102. doi: 10.1093/gigascience/giae102 (PMC11659977; doi:10.1093/gigascience/giae102)
Supplement: giae102_Response_to_Reviewer_Comments_Revision_2 [file giae102_response_to_reviewer_comments_revision_2.pdf]

Author's Response To Reviewer Comments

We thank the remaining reviewer as well as the editor for their interest in our work and positive comments on the manuscript. We appreciate the opportunity to again resubmit the manuscript and agree that it has improved our manuscript through these rounds of revisions.

Reviewer reports:

> Reviewer #1: The authors have provided example code and tutorials on the
> cfDNA UniFlow GitHub page, which is excellent. I would like to address the
> authors by stating that my previous two responses were focused on the
> reliability of functions in cfDNA UniFlow. As a tool designed to
> preprocess multiple cfDNA sequencing data and highlight this capability,
> the previous benchmarking using only three samples and conducting only
> TF-related analyses was indeed unconvincing. This is why I consistently
> requested the authors to increase the scope of benchmarking. After these
> revisions, which included the addition of dataset from Snyder et al. and
> the WPS analysis, the overall analytical capability of cfDNA UniFlow is
> now more convincing. However, it should be noted that the dataset from
> Snyder et al. was not mentioned in the Data Availability or
> Acknowledgements.

In the previous version of the manuscript, we referenced data used for supplementary analysis in the supplement and only cited Snyder et al. in the main text. As response to this request, we added the dataset reference to the Data Availability section of the main text. The new section reads as follows:

The data used for generating plots included in this article are available in the European Genome-Phenome Archive at https://ega-archive.org with accession EGAD00001010100. The aligned reads from the Snyder et al. Cell 2016, which were used for Supplementary Figures 8-10, are deposited in the NCBI Gene Expression Omnibus (GEO) with accession GSE71378 and can be downloaded via the Sequence Read Archive (SRA). Files used for testing cfDNA UniFlow are available on Zenodo (DOI: 10.5281/zenodo.13768203).

> Additionally, the authors should provide an end-to-end demo. As I have
> mentioned multiple times, cfDNA UniFlow emphasizes its advantages as a
> preprocessing pipeline, yet it lacks an end-to-end demo from raw data to
> report. The three demo datasets provided by the authors (GitHub sections
> 3.2, 3.3, and 3.4) all start the analysis from BAM files. The author may
> consider replacing the BAM file used in Section 3.2 with a FASTQ file, as
> the processing workflow for the vast majority of cfDNA sequencing data
> typically starts with FASTQ files.

Previous test-sets were converted to FASTQ files and uploaded to the Webserver and to Zenodo. We added two new configuration files and the corresponding documentation for running the integration test starting from FASTQ files. These configuration files are further referenced in subsequent sections as a reference for configuring new experiments.

> Additionally, the data download link provided in Section 3.2
> (https://kircherlab.bihealth.org/download/cfDNA-testSam) still requires
> authentication. Please check this issue.

Apparently, there was a confusion regarding the link mentioned in the description text. The actual link used for downloading in the code snippet was working properly. We fixed the truncated link in the textual description, which due to its truncation resulted in a user authentication.
