# Supplementary material for: cfDNA UniFlow: a unified preprocessing pipeline for cell-free DNA data from liquid biopsies
Source: Gigascience. 2024 Dec 20;13:giae102. doi: 10.1093/gigascience/giae102 (PMC11659977; doi:10.1093/gigascience/giae102)
Supplement: giae102_Reviewer_1_Report_Original_Submission — Wei Zhang -- 12/18/2023 Reviewed [file giae102_reviewer_1_report_original_submission.pdf]

Reviewer Report

Title: cfDNA UniFlow: A unified preprocessing pipeline for cell-free DNA data from liquid biopsies

Version: Original Submission **Date:** 12/18/2023

Reviewer name: Wei Zhang

Reviewer Comments to Author:

In this study, the authors proposed a new cfDNA sequencing data processing pipeline named "cfDNA UniFlow". this toolkit is based on snakemake and can be installed through conda. cfDNA UniFlow includes the necessary data pre-processing steps as well as downstream tasks like fragment-length analysis and GC bias correction. The manuscripts are well-written, but there are some issues that need to be addressed.Major:1. The current functionality of cfDNA UniFlow appears overly basic, with downstream feature extraction and analysis being relatively simplistic. The review article "Epigenetics, fragmentomics, and topology of cell-free DNA in liquid biopsies," published in Science, provides a comprehensive summary of features related to cfDNA sequencing data, including DNA methylation, CNVs, mutations, and the ratio of short to long fragments, as well as oriented end density. In 2021, Zhang and colleagues developed cfDNApipe, which includes modules for extracting genomic, methylomic, and fragmentomic information. I recommend that the authors enhance the downstream analysis capabilities of cfDNA UniFlow and conduct a comparative analysis with cfDNApipe.2. In the manuscript, the authors should incorporate a more detailed presentation of analytical results, particularly for each downstream task using various real datasets. Additionally, on the GitHub page, the authors should provide reproducible data and analysis workflows. For instance, while the cfDNA UniFlow GitHub page outlines installation and testing procedures, these instructions are not sufficiently detailed. For example, in Section "Obtain a copy of this workflow," providing direct execution codes would facilitate quicker installation and deployment for users. Moreover, I encountered errors when attempting to download test data from the webserver provided by the authors (Section 3.2 Integration Test, Step 1: Download test files). For instance, using curl to download results in an error message, "Failed to open the file resources/testsample/testsample_hg19_1x_chr20-22.bam: No such file or directory." Also, clicking on the webserver hyperlink prompts for a username and password, preventing me from conducting tests.Therefore, I think that cfDNA UniFlow still requires modifications in terms of functionality and user guidance.

Level of Interest

Please indicate how interesting you found the manuscript: Choose an item.

Quality of Written English

Please indicate the quality of language in the manuscript: Choose an item.

Declaration of Competing Interests

Please complete a declaration of competing interests, considering the following questions:

- Have you in the past five years received reimbursements, fees, funding, or salary from an organisation that may in any way gain or lose financially from the publication of this manuscript, either now or in the future?
- Do you hold any stocks or shares in an organisation that may in any way gain or lose financially from the publication of this manuscript, either now or in the future?
- Do you hold or are you currently applying for any patents relating to the content of the manuscript?
- Have you received reimbursements, fees, funding, or salary from an organization that holds or has applied for patents relating to the content of the manuscript?
- Do you have any other financial competing interests?
- Do you have any non-financial competing interests in relation to this paper?

If you can answer no to all of the above, write 'I declare that I have no competing interests' below. If your reply is yes to any, please give details below.

I declare that I have no competing interests.

I agree to the open peer review policy of the journal. I understand that my name will be included on my report to the authors and, if the manuscript is accepted for publication, my named report including any attachments I upload will be posted on the website along with the authors' responses. I agree for my report to be made available under an Open Access Creative Commons CC-BY license (http://creativecommons.org/licenses/by/4.0/). I understand that any comments which I do not wish to be included in my named report can be included as confidential comments to the editors, which will not be published.

Choose an item.

To further support our reviewers, we have joined with Publons, where you can gain additional credit to further highlight your hard work (see: https://publons.com/journal/530/gigascience). On publication of this paper, your review will be automatically added to Publons, you can then choose whether or not to claim your Publons credit. I understand this statement.

Yes Choose an item.
