# Supplementary material for: cfDNA UniFlow: a unified preprocessing pipeline for cell-free DNA data from liquid biopsies
Source: Gigascience. 2024 Dec 20;13:giae102. doi: 10.1093/gigascience/giae102 (PMC11659977; doi:10.1093/gigascience/giae102)
Supplement: giae102_Reviewer_1_Report_Revision_1 — Wei Zhang -- 6/6/2024 Reviewed [file giae102_reviewer_1_report_revision_1.pdf]

Reviewer Report

Title: cfDNA UniFlow: A unified preprocessing pipeline for cell-free DNA data from liquid biopsies

Version: Revision 1 **Date:** 6/6/2024

Reviewer name: Wei Zhang

Reviewer Comments to Author:

The authors' response addressed some questions, but some replies did not resolve my concerns. My major comments still focus on the issues previously mentioned, while the minor comments only pertain to some writing issues.MajorMy concern mainly centers on the validity of the results and the usability of the methods.1. Preprocessing is important, but it must be validated in conjunction with downstream analysis. Regarding the previous issue of "reproducible data and analysis workflows", I intended for the authors to provide more analysis results based on cfDNA UniFlow to highlight the pipeline's advantages and validity in data preprocessing. Compared to other papers published in GigaScience, cfDNA UniFlow only provides one TF analysis result involving three samples in the main text. A pipeline is a fundamental work, and without support from various downstream analyses, this does not effectively demonstrate the validity of this work. Additionally, the data used in this paper is stored in EGA. As the authors mentioned, this data requires permission for download. However, some cfDNA sequencing data can be downloaded directly. For example, Snyder et al.'s article published in CELL provides sequencing data for multiple cancer types, indexed as GSE71378. This data is publicly downloadable, is substantial in volume, and has been used in multiple studies. I also suggest that the authors search for other pipeline-related TECHNICAL NOTES in GigaScience to see how other papers present their results and add validity verification from different data sources based on the current foundation.2. Regarding the usability of the methods, the authors' updates on the examples in GitHub are excellent. The tutorial provided for secondary development based on this is quite detailed. The authors stated in their response, "as we expect that users will add their own modules and extensions of the workflow, we suggest an alternative to the fork process rather than a simple clone." For this cfDNA preprocessing pipeline, the most crucial aspect is ensuring user usability. For most groups capable of independently developing algorithms, building their own cfDNA processing pipeline or integrating their algorithms into the downstream of cfDNA UniFlow would not be difficult.However, many researchers interested in cfDNA-related studies are biologists and clinicians who often prefer to use the functions and obtain solid results. They might not be very familiar with GitHub commands or Snakemake. Therefore, I suggest that the authors provide specific and basic download commands and give some detailed explanations and instructions for the result files. Here are some examples based on the specific functions already implemented in cfDNA UniFlow to help the authors make modifications:-How to run a specific function like CNV analysis independently (to facilitate users in invoking functions independently)-The file storage structure for all results, whether they are distinguished by name or by folder. An introduction to some important files (to help users locate the results of each function).-How to check log files after a program interruption (to help users debug)-How users can adjust parameters such as alignment (to enable users to customize cfDNA UniFlow settings)-How to dynamically skip certain analyses that users do not want to perform (to

allow users to customize the pipeline)Minor1. The title includes a description of the data type, and I suggest the authors re-consider it. As it stands, cfDNA UniFlow primarily handles cfDNA sequencing data, not involving cfDNA methylation or other sequencing data, while the term "DNA data" is too broad.2. The authors provided a detailed functional comparison with cfDNApipe in their response, which clearly demonstrates the advantages of cfDNA UniFlow. I suggest including this table in the main text to replace Fig. 2. The current Fig. 2 in the main text can be moved to the supplementary materials.3. The text mentions, "Additionally, this pipeline does not address issues of batch and bias correction, which might be most relevant in such aggregation efforts." The batch issue is a very challenging problem for all bioinformatics analysis algorithms, and cfDNA UniFlow does not address the batch problem. I suggest modifying this statement.

Level of Interest

Please indicate how interesting you found the manuscript: Choose an item.

Quality of Written English

Please indicate the quality of language in the manuscript: Choose an item.

Declaration of Competing Interests

Please complete a declaration of competing interests, considering the following questions:

- Have you in the past five years received reimbursements, fees, funding, or salary from an organisation that may in any way gain or lose financially from the publication of this manuscript, either now or in the future?
- Do you hold any stocks or shares in an organisation that may in any way gain or lose financially from the publication of this manuscript, either now or in the future?
- Do you hold or are you currently applying for any patents relating to the content of the manuscript?
- Have you received reimbursements, fees, funding, or salary from an organization that holds or has applied for patents relating to the content of the manuscript?
- Do you have any other financial competing interests?
- Do you have any non-financial competing interests in relation to this paper?

If you can answer no to all of the above, write 'I declare that I have no competing interests' below. If your reply is yes to any, please give details below.

I declare that I have no competing interests

I agree to the open peer review policy of the journal. I understand that my name will be included on my report to the authors and, if the manuscript is accepted for publication, my named report including any

attachments I upload will be posted on the website along with the authors' responses. I agree for my report to be made available under an Open Access Creative Commons CC-BY license (http://creativecommons.org/licenses/by/4.0/). I understand that any comments which I do not wish to be included in my named report can be included as confidential comments to the editors, which will not be published.

Choose an item.

To further support our reviewers, we have joined with Publons, where you can gain additional credit to further highlight your hard work (see: https://publons.com/journal/530/gigascience). On publication of this paper, your review will be automatically added to Publons, you can then choose whether or not to claim your Publons credit. I understand this statement.

Yes Choose an item.
