# Supplementary material for: cfDNA UniFlow: a unified preprocessing pipeline for cell-free DNA data from liquid biopsies
Source: Gigascience. 2024 Dec 20;13:giae102. doi: 10.1093/gigascience/giae102 (PMC11659977; doi:10.1093/gigascience/giae102)
Supplement: giae102_Reviewer_2_Report_Original_Submission — Zsolt BalÃ¡zs, PhD, MD -- 2/28/2024 Reviewed [file giae102_reviewer_2_report_original_submission.pdf]

Reviewer Report

Title: cfDNA UniFlow: A unified preprocessing pipeline for cell-free DNA data from liquid biopsies

Version: Original Submission **Date:** 2/28/2024

Reviewer name: Zsolt BalÃ¡zs, PhD, MD

Reviewer Comments to Author:

RÃ¶ner et al. present a cfDNA Uniflow, a standardized, ready to use workflow for processing cfDNA samples. The software they describe is available from GitHub with a documentation including installation and usage. The workflow is relevant to the field, however offers similar results albeit with fewer functionality than the workflow cfDNApipe published by Zhang et al. in 2021. While the Technical Note does a good job describing the structure of the software tool, it is unclear in what way cfDNA Uniflow is novel and an improvement over the predecessor published 3 years ago. My main comments center around this issue, as I feel that this is crucial for the publication of the manuscript.Major comments:- The authors make mention of an earlier workflow cfDNApipe writing: "The utility functions range from generation of summary statistics, GC-bias correction tailored towards CNV detection and extraction of a limited number of features [24]." The authors then present their workflow which performs the same preprocessing steps using updated algorithms (e.g. bwa-mem2 instead of bowtie for mapping), however only offer two downstream feature extraction methods (copy number analysis CNA, and comparing coverage at regions of interest ~ nucleosome footprints across samples), compared to the multitude of analyses offered by cfDNApipe. Does cfDNA Uniflow offer added value other than the use of more contemporary software versions?- Given that the workflow is written in Snakemake the Introduction section could also mention other Snakemake-implemented cfDNA workflows such as cfCloud https://www.sciencedirect.com/science/article/pii/S0006497118726487 or cfdna https://github.com/jeszyman/cfdna and describe how the current workflow improves upon those.- The authors criticize cfDNApipe in the Introduction section writing that its "design does not allow for easy integration of new functionalities, creating the need for either an additional workflow management system or extensive modification of the original code". In what way is cfDNA Uniflow overcoming this issue?- Figure 1 depicts three different downstream analysis methods (four if we count CNA): nucleosome footprinting, fragment end motifs and fragment length analysis, however, in the workflow only nucleosome footprinting is implemented (and CNA). It might be advisable to indicate in the figure, that these last two steps are not implemented in the workflow whereas the rest of the figure depicts steps all implemented within the workflow.- The authors highlight the effects of their own GC-bias correction method (Figure 3), however this GC-bias correction method is only linked and briefly described in the Signal Extraction subsection, even though the tool has as of yet not been peer reviewed. Considering the limited number of novel functions in this workflow, the novel GC-bias correction method is a key added value. The description of a novel GC-bias correction method and its benchmarking against existing methods, alongside a demonstration of its utility in tasks other than nucleosome footprinting (e.g. its effects on CNA) would greatly increase the value of the manuscript. The GC-bias correction is left as a black box when it could be a selling feature of the workflow.Minor

comments:- What is the rationale behind treating CNA as "utility function", but nucleosome footprinting as "downstream analysis" in Figure 1?- It feels like the title "... pipeline for cell-free DNA from liquid biopsies" is missing a word after cfDNA (sequencing data?).- In the Preprocessing section, it is unclear whether the adapter removal is only done by Trimmomatic when there is no need to merge the fastq files by NGmerge or whether even after merging, the users can opt to use Trimmomatic instead.- Some typos appear to have evaded proof-reading: e.g. Implementation section "We implemented in the cfDNA UniFlow workflow in the popular workflow management system Snakemake [25]."- Some formulations seem imprecise to me:- "calculating (?) all possible fragments for a specified range of fragment lengths" - maybe counting instead of calculating?- "and GRHL2, a pioneer (?) TF for epithelial cells [34] playing a role in a wide variety of cancer types [35-39]." - maybe signature instead of pioneer?

Level of Interest

Please indicate how interesting you found the manuscript: Choose an item.

Quality of Written English

Please indicate the quality of language in the manuscript: Choose an item.

Declaration of Competing Interests

Please complete a declaration of competing interests, considering the following questions:

- Have you in the past five years received reimbursements, fees, funding, or salary from an organisation that may in any way gain or lose financially from the publication of this manuscript, either now or in the future?
- Do you hold any stocks or shares in an organisation that may in any way gain or lose financially from the publication of this manuscript, either now or in the future?
- Do you hold or are you currently applying for any patents relating to the content of the manuscript?
- Have you received reimbursements, fees, funding, or salary from an organization that holds or has applied for patents relating to the content of the manuscript?
- Do you have any other financial competing interests?
- Do you have any non-financial competing interests in relation to this paper?

If you can answer no to all of the above, write 'I declare that I have no competing interests' below. If your reply is yes to any, please give details below.

I declare that I have no competing interests.

I agree to the open peer review policy of the journal. I understand that my name will be included on my report to the authors and, if the manuscript is accepted for publication, my named report including any

attachments I upload will be posted on the website along with the authors' responses. I agree for my report to be made available under an Open Access Creative Commons CC-BY license (http://creativecommons.org/licenses/by/4.0/). I understand that any comments which I do not wish to be included in my named report can be included as confidential comments to the editors, which will not be published.

Choose an item.

To further support our reviewers, we have joined with Publons, where you can gain additional credit to further highlight your hard work (see: https://publons.com/journal/530/gigascience). On publication of this paper, your review will be automatically added to Publons, you can then choose whether or not to claim your Publons credit. I understand this statement.

Yes Choose an item.
